# Clonal dynamics of aggressive systemic mastocytosis on avapritinib therapy

**DOI:** 10.1038/s41408-024-01157-w

**Published:** 2024-10-14

**Authors:** Xiaomeng Huang, Anthony D. Pomicter, Jonathan Ahmann, Yi Qiao, Opal S. Chen, Tracy I. George, Nataly Cruz-Rodriguez, Sameer Ahmad Guru, Gabor T. Marth, Michael W. Deininger

**Affiliations:** 1 Utah Center for Genetic Discovery, University of Utah, Salt Lake, UT USA; 2 Department of Human Genetics, School of Medicine, University of Utah, Salt Lake, UT USA; 3 Division of Hematology Biorepository, Huntsman Cancer Institute, University of Utah, Salt Lake, UT USA; 4 High-throughput Genomics, Huntsman Cancer Institute, University of Utah, Salt Lake, UT USA; 5 Department of Pathology, School of Medicine, University of Utah, Salt Lake, UT USA; 6 ARUP Laboratories, Salt Lake, UT USA; 7 Versiti Blood Research Institute, Milwaukee, WI USA

**Keywords:** Cancer genomics, Haematological cancer

Advanced systemic mastocytosis (AdvSM) is characterized by the expansion of clonal mast cells (MCs) in extracutaneous tissues such as bone marrow (BM) or liver, with consecutive organ damage [1]. Some AdvSM cases are limited to the MC lineage, but many are associated with a myeloid hematologic neoplasm (ASM-AHN), most commonly chronic myelomonocytic leukemia (CMML). The *KIT* D816V mutation (*KIT*^*D816V*^) is present in >90% of ASM-AHN cases and thought to be the main disease driver [2]. *KIT* is expressed by normal hematopoietic stem and progenitor cells and promotes MC differentiation [3]. An MC component is present in the BM of many patients with *KIT*^*D816V*+^ myeloid neoplasms other than AdvSM [4]. Conversely, *KIT*^*D816V*^ was detected in the AHN component of >90% of ASM-CMML [5]. Avapritinib, a potent and selective KIT^D816V^ inhibitor, demonstrated a 75% overall response rate (ORR) per modified mIWG-MRT-ECNM criteria in the phase 1 EXPLORER (NCT02561988) study and the phase 2 PATHFINDER (NCT03580655) study [6, 7]. Responses were mostly durable and occurred across AdvSM subtypes, leading to FDA and EMA regulatory approval.

ASM-AHN patients regularly have mutations in genes other than *KIT*, and those in *SRSF2*, *ASXL1*, and *RUNX1* are associated with poor outcome [8]. Given their complex clonal relationships, predicting how MC and AHN components respond to *KIT*^*D816V*^ inhibition is not straightforward. However, it is plausible that *KIT*^*D816V*^*’s* position in the clonal architecture governs response. To start addressing this question, we mapped clonal architecture in four ASM-AHN patients treated with avapritinib, combining whole genome sequencing (WGS), colony sequencing, and single-cell RNA sequencing (scRNAseq).

## Mutational landscape and cell populations

We first charted the mutational landscape prior to avapritinib treatment by 120x WGS of white blood cells, using skin fibroblasts as controls. We detected an average of 1891 somatic mutations, including mutations in *KIT*, *TET2*, *TP53*, *SRSF2*, *CUX1*, *ETNK1*, *ASXL1*, *EZH2*, and *KDM6A* (details: Supplementary Materials). Although all patients were *KIT*^*D816V+*^ by ddPCR, WGS was negative in Pt1, suggesting <1% *KIT*^*D816V*+^ cells. Copy number alterations were rare. Pt1 had a clonal homozygous deletion on chromosome 21 (*RUNX1*). Subclonal LOH was observed in Pt2 (chromosome 7q), Pt3 (chromosome 4q), and Pt4 (chromosomes 7q and 21) (Supplementary Fig. 1A–D). Mutation types were similar across patients and dominated by C > T transitions (Supplementary Fig. 2A, B).

ASM-AHN are complex myeloid neoplasms where malignant and normal myeloid cells co-exist with immune cells. To understand the cellular composition of this ecosystem, we performed scRNAseq on the four ASM-AHN patients, using healthy donors, and three untreated *KIT*^*WT*^ CMML patients for comparison. We identified twelve distinct clusters (C1–C12, Supplementary Fig. 3A, B). B cells (C1), T cells (C2), NK cells (C3), red blood cells (C7), platelets (C8), and plasma cells (C9) overlapped between controls and disease states. In contrast, monocytes (C4) from each patient formed a unique cluster distinct from healthy donors. Mature neutrophils (C5) and basophils (C10) were present in both controls and patients, while immature neutrophils (C6) were only found in patients (Supplementary Fig. 3C–E). C11 cells, enriched in Pt4 and Pt3, expressed *CD34* and genes associated with mature eosinophils (*LAIR1*, *ITGA4*, *IL3RA*) and MCs (*KIT*, *TPSAB1*, *CPA3*) (Supplementary Fig. 3F), suggesting origin from a progenitor committed to eosinophil-MC differentiation, consistent with Pt4’s ASM-chronic eosinophilic leukemia diagnosis [9]. In contrast, Pt3 had ASM-myelodysplastic syndrome (MDS)/myeloproliferative neoplasm (MPN)-U with Grade 2-3 fibrosis without eosinophilia. Pt3’s C11 cells lacked *IL3RA*, suggesting a closer relation to MCs than eosinophil precursors. These findings align with reports of *KIT*^*D816V*^ in blood eosinophils of >50% of SM patients and suggest MC and eosinophil lineages are closely related [10]. Lastly, C12 cells, present mainly in Pt1 and CMML3, exhibited mesenchymal (collagen), inflammatory (TIMP1), and phagocytic (osteopontin) features (Supplementary Fig. 3G) [11]. Both patients had aggressive disease. Pt1 deteriorated clinically with refractory ascites despite sustained reduction of tryptase on avapritinib (Supplementary Fig. 1). CMML3 rapidly progressed to AML on 5-azacytidine. These cells may originate from a remodeled BM niche [12], consistent with Pt1’s severe fibrosis. A previous study failed to detect CD45^−^CD34^−^CD105^++^CD73^+^ mesenchymal stromal cells (MSC) in the blood of myelofibrosis patients [13]. Our findings suggest that MSCs may be present but exhibit an aberrant immunophenotype.

## Clonal and cell population dynamics on avapritinib

**Fig. 1:**
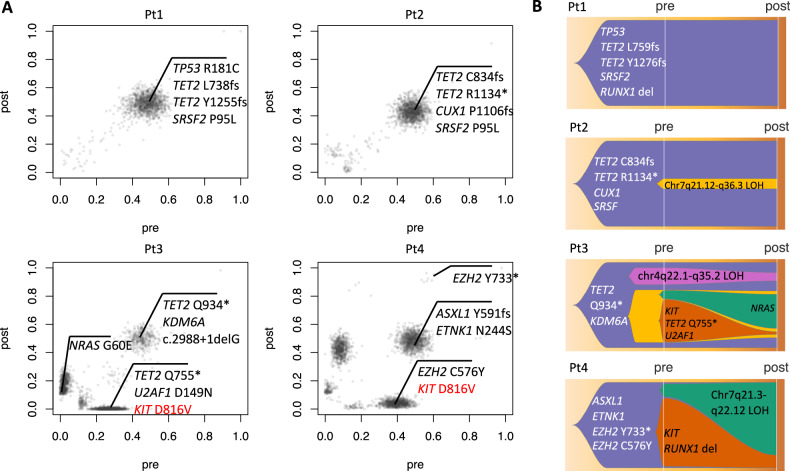
Clonal dynamics on avapritinib therapy. **A** Variant allele frequencies of somatic mutations detected by WGS in pre- and post-therapeutic samples were plotted against each other. **B** Fish plots show the subclone dynamics for pre- and post-therapeutic samples. Driver mutations and CNVs are labeled in each subclone.

We combined WGS and scRNAseq data obtained at several time points for each pt to assess the effect of avapritinib on clonal architecture and cellular ecosystems (Fig. 1 and Supplementary Fig. 1). Subclone structures were established using SubcloneSeeker [14], which outputs the mostly likely clonal architecture based on VAF. Pt1 had one stable clone, and Pt2 had a small subclone with chromosome 7q21.12–q36.3 LOH that remained unchanged, consistent with undetectable or low *KIT*^*D816V*^ blood levels. A different pattern was observed in Pt3 and Pt4, where avapritinib suppressed the *KIT*^*D816V*+^ clone in the blood and eliminated abnormal BM MCs. While *KIT*^*D816V*^ subclones decreased, subclones with other driver mutations persisted. Specifically, Pt3’s founder clone (SC1) with *TET2*^Q934*^ and *KDM6A* mutations gave rise to two subclones, SC5 and SC2. SC2 further evolved into SC3 with *TET2*^Q755*^ and *KIT*^*D816V*^, which was reduced below WGS detection limit on avapritinib and SC4 with *NRAS*^*G60E*^ (Supplementary Fig. 4A, B and Fig. 1B). Interestingly, although SC5 was expected to inherit *TET2*^Q934*^ from the founder clone, *TET2* was wild type due to chromosome 4q22.1–q35.2 LOH, validated by colony sequencing (Supplementary Fig. 5). In Pt4, the founder clone (SC1) had *ASXL1*, *ETNK1*, and *EZH2* mutations. On therapy, a large subclone (SC2) with *KIT*^*D816V*^ and *RUNX1* deletion contracted from ~80% to ~10%, while a subclone (SC3) with chromosome 7q21.3-q22.12 LOH expanded from ~15% to ~85% (Supplementary Fig. 4C, D and Fig. 1B). Although *EZH2*^Y733*^ and *EZH2*^*C576Y*^ are in the founder clone SC1 because *EZH2* is located within the chromosome 7q21.3-q22.12 LOH region, both mutations had a large variant allele frequency shift between pre- and post-treatment samples due to the expansion of subclone SC3 that contains this LOH event (Fig. 1A, B). For validation of clonal structure, we genotyped single granulocyte-macrophage colonies derived from CD34^+^ cells. Only pretreatment samples from Pts 1, 3, and 4 grew colonies. We confirmed driver mutations (e.g., in *TET2*, *TP53*, and *SRSF2* in Pt2, and *ETNK1*, *ASXL1*, and *EZH2* in Pt4), and a subset of subclones defined by WGS data (SC5 in Pt3, and SC3 in Pt4) (Supplementary Materials).

Next, we compared scRNAseq data between pre- and post-treatment samples in each patient (Supplementary Fig. 6A, B). While no significant changes were seen in B, T, NK cells and mature neutrophils (Supplementary Fig. 7A–D), avapritinib reduced the CD34^+^/eosinophil/MC population in Pt3 and Pt4 (Supplementary Fig. 7F and Supplementary Table 1) and decreased collagen-enriched putative MSCs in Pt1 (Supplementary Fig. 7G and Supplementary Table 1). Immature neutrophils decreased on avapritinib in Pt1, Pt2, and Pt4 (Supplementary Fig. 7H and Supplementary Table 1). Monocytes in Pt3 and Pt4 remained similar to healthy controls, while those in Pt1 and Pt2 became more similar to controls (Supplementary Fig. 7E, Supplementary Materials).

**Fig. 2:**
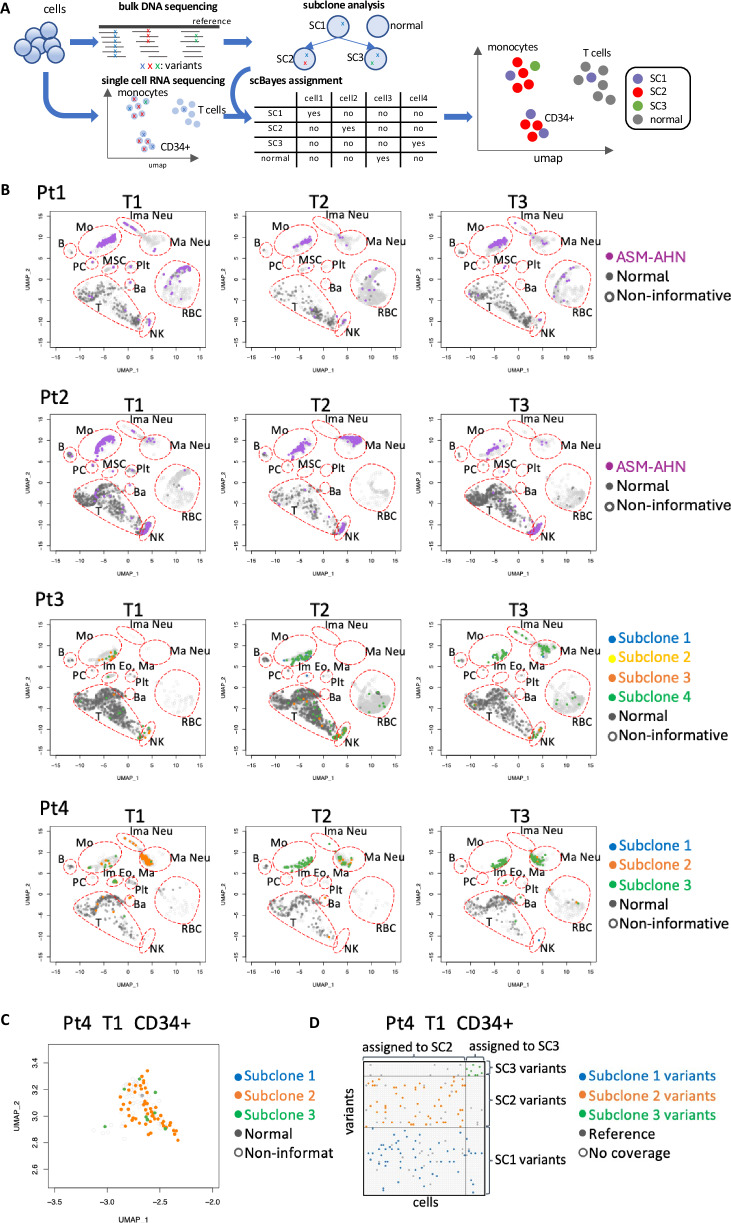
Clonal attribution by scBayes. **A** Schematic of scBayes algorithm. The scBayes method integrates bulk DNA sequencing-based subclone analysis and single-cell RNA sequencing-based transcriptomic data to determine the subclone identity of individual cells. **B** Umap plots of clonal attribution by scBayes across all patients (Pt1-4). Circles represent non-informative cells, while gray dots represent normal cells. For Pt1 and Pt2, purple dots correspond to ASM-AHN cells. For Pt3 and Pt4, blue, orange, and green dots represent cells from different subclones. **C** UMAP plot of *CD34*^+^ cells with subclone identities for Pt4. **D** Clonal attribution by scBayes to *CD34*^+^ cells for Pt4. The *x-* axis corresponds to individual cells, and the y-axis to individual variants. A cell-variant coordinate is filled in when sequencing coverage is detected in that cell at the variant’s location. The color corresponds to different subclone-defining variants.

To attribute individual cells to specific subclones, we used scBayes, a Bayesian framework based on cross-referencing somatic mutations detected by scRNAseq and WGS (Fig. 2A) [15]. Approximately 32% of monocytes, 7% of neutrophils, and 46% of CD34^+^ cells were informative (Supplementary Tables 2–6 and Fig. 2B). Only ~1% of myeloid cells were “normal” (i.e., containing no somatic mutations), and this proportion did not increase on treatment. CD34^+^ cells were exclusively clonal and included both evolutionary branches in Pt4 (Fig. 2C, D). Shifts in clonal architecture were reflected in cell populations in Pt3 and Pt4: “orange” cells from a *KIT*^*D816V+*^ subclone were replaced by “green” cells from *KIT*^*WT*^ subclones on avapritinib (Fig. 2B). Approximately 43% of lymphocytes were informative, with ~5% attributed to ASM-AHN (Supplementary Table 2), indicating most lymphocytes are not part of the malignant clone. Notably, 48% of NK cells were clonal, compared to 2% of T cells and 4% of B cells, reflecting either their short life span or origin from a myeloid progenitor cell, as previously reported [16].

Depending on *KIT*^*D816V*^’s position in the clonal architecture, targeting this driver may differentially affect cellular compartments contributing to ASM-AHN. There are three possible scenarios: (1) if *KIT*^*D816V*^ is the founder mutation, avapritinib should target all abnormal cells and promote re-establishment of normal hematopoiesis, similar to imatinib in chronic phase chronic myeloid leukemia [17]. In our four ASM-AHN cases, we did not observe the emergence of normal myeloid cells during treatment, suggesting *KIT*^*D816V*^ is not the founder mutation. However, it is important to consider the small sample size and that nonclonal recovery in hematopoietic stem cells remains possible. (2) If *KIT*^*D816V*^ was acquired on the background of other driver mutations but prior to the branching of MC and AHN components, avapritinib should target both. Results from Pt3 and Pt4 support this scenario, as avapritinib eliminated abnormal BM MCs and suppressed *KIT*^*D816V+*^ clones in the blood. (3) If *KIT*^*D816V*^ was acquired after the branching, avapritinib should selectively affect the *KIT*^*D816V*^ subclone, including its MC component. This is represented in Pt1 and Pt2, where avapritinib reduced or eliminated abnormal BM MCs, while abnormal myeloid cells persisted in the blood. Three out of four patients had *TET2* mutations in the founder clone, suggesting hypomethylating agents like 5-azacytidine could be combined with avapritinib to address the entire malignant network and promote normal hematopoiesis. Altogether, our data suggest clonal dynamics in AdvSM are influenced by multiple factors, including genotype, driver mutation position, and tissue environment. ASM-AHN should be seen as a complex myeloid neoplasm with a significant MC component. High-resolution mapping of this ecosystem may reveal strategies to rebalance the system beyond targeting *KIT*^*D816V+*^ clones.

## Supplementary information

Suppl Material

Suppl Figures

Suppl Table 1 Cell count per cell type

Suppl Table 2 Informative, non-informative and proportion of clonally attributable cells by cell type

Suppl Table 3 scBayes assignment for Pt1

Suppl Table 4 scBayes assignment for Pt2

Suppl Table 5 scBayes assignment for Pt3

Suppl Table 6 scBayes assignment for Pt4

Suppl Table 7 Pathology, Cytogenetics, and Next Generation Sequencing

Suppl Table 8 Primers for sequencing colonies

Suppl Table 9 Markers for cell type identification

## Data Availability

The scRNAseq gene expression data is available at the Gene Expression Omnibus with accession number GSE249445. The custom scripts and processed datasets generated and/or analyzed in the study, as well as somatic mutations from 4 ASM-AHN patients, are available in the GitHub repository https://github.com/xiaomengh/asm-analysis-2023.

## References

[1] Valent P, Hartmann K, Hoermann G, Reiter A, Alvarez-Twose I, Brockow K, et al. Harmonization of diagnostic criteria in mastocytosis for use in clinical practice: WHO vs ICC vs AIM/ECNM. J Allergy Clin Immunol Pract. 2024, 10.1016/j.jaip.2024.08.044. 10.1016/j.jaip.2024.08.044 39216803

[2] Kitayama H, Tsujimura T, Matsumura I, Oritani K, Ikeda H, Ishikawa J, et al. Neoplastic transformation of normal hematopoietic cells by constitutively activating mutations of c-kit receptor tyrosine kinase. Blood. 1996;88:995–1004. 8704259

[3] Tsai M, Shih LS, Newlands GF, Takeishi T, Langley KE, Zsebo KM, et al. The rat c-kit ligand, stem cell factor, induces the development of connective tissue-type and mucosal mast cells in vivo. Analysis by anatomical distribution, histochemistry, and protease phenotype. J Exp Med. 1991;174:125–31. 1711559 10.1084/jem.174.1.125 PMC2118877

[4] Craig JW, Hasserjian RP, Kim AS, Aster JC, Pinkus GS, Hornick JL, et al. Detection of the KIT(D816V) mutation in myelodysplastic and/or myeloproliferative neoplasms and acute myeloid leukemia with myelodysplasia-related changes predicts concurrent systemic mastocytosis. Mod Pathol. 2020;33:1135–45. 31896808 10.1038/s41379-019-0447-x

[5] Sotlar K, Colak S, Bache A, Berezowska S, Krokowski M, Bültmann B, et al. Variable presence of KITD816V in clonal haematological non-mast cell lineage diseases associated with systemic mastocytosis (SM-AHNMD). J Pathol 2010;220:586–95. 20112369 10.1002/path.2677

[6] Gotlib J, Reiter A, Radia DH, Deininger MW, George TI, Panse J, et al. Efficacy and safety of avapritinib in advanced systemic mastocytosis: interim analysis of the phase 2 PATHFINDER trial. Nat Med. 2021;27:2192–9. 34873345 10.1038/s41591-021-01539-8 PMC8674139

[7] DeAngelo DJ, Radia DH, George TI, Robinson WA, Quiery AT, Drummond MW, et al. Safety and efficacy of avapritinib in advanced systemic mastocytosis: the phase 1 EXPLORER trial. Nat Med. 2021;27:2183–91. 34873347 10.1038/s41591-021-01538-9 PMC8674134

[8] Jawhar M, Schwaab J, Schnittger S, Meggendorfer M, Pfirrmann M, Sotlar K, et al. Additional mutations in SRSF2, ASXL1 and/or RUNX1 identify a high-risk group of patients with KIT D816V(+) advanced systemic mastocytosis. Leukemia. 2016;30:136–43. 26464169 10.1038/leu.2015.284

[9] Drissen R, Thongjuea S, Theilgaard-Mönch K, Nerlov C. Identification of two distinct pathways of human myelopoiesis. Sci Immunol. 2019;4:eaau7148. 31126997 10.1126/sciimmunol.aau7148

[10] Navarro‐Navarro P, Álvarez‐Twose I, Pérez‐Pons A, Henriques A, Mayado A, García‐Montero AC, et al. KITD816V mutation in blood for the diagnostic screening of systemic mastocytosis and mast cell activation syndromes. Allergy. 2023;78:1347–59. 36385619 10.1111/all.15584

[11] Eckfeld C, Schoeps B, Häußler D, Frädrich J, Bayerl F, Böttcher JP, et al. TIMP-1 is a novel ligand of amyloid precursor protein and triggers a proinflammatory phenotype in human monocytes. J Cell Biol. 2023;222:e202206095. 36629908 10.1083/jcb.202206095 PMC9837626

[12] Schepers K, Pietras EM, Reynaud D, Flach J, Binnewies M, Garg T, et al. Myeloproliferative neoplasia remodels the endosteal bone marrow niche into a self-reinforcing leukemic niche. Cell Stem Cell. 2013;13:285–99. 23850243 10.1016/j.stem.2013.06.009 PMC3769504

[13] de la Guardia RD, Correa JG, López‐Millán B, Juan M, Bueno C, Cervantes F, et al. Detection of inflammatory monocytes but not mesenchymal stem/stromal cells in peripheral blood of patients with myelofibrosis. Br J Haematol. 2018;181:133–7. 28220930 10.1111/bjh.14507

[14] Qiao Y, Quinlan AR, Jazaeri AA, Verhaak RG, Wheeler DA, Marth GT. SubcloneSeeker: a computational framework for reconstructing tumor clone structure for cancer variant interpretation and prioritization. Genome Biol. 2014;15:443. 25160522 10.1186/s13059-014-0443-x PMC4180956

[15] Qiao Y, Huang X, Moos PJ, Ahmann JM, Pomicter AD, Deininger MW, et al. A Bayesian framework to study tumor subclone-specific expression by combining bulk DNA and single-cell RNA sequencing data. Genome Res. 2024;34:94–105. 38195207 10.1101/gr.278234.123 PMC10903947

[16] Chen Q, Ye W, Jian Tan W, Mei Yong KS, Liu M, Qi Tan S, et al. Delineation of natural killer cell differentiation from myeloid progenitors in human. Sci Rep. 2015;5:15118. 26456148 10.1038/srep15118 PMC4600975

[17] Bumm T, Müller C, Al-Ali HK, Krohn K, Shepherd P, Schmidt E, et al. Emergence of clonal cytogenetic abnormalities in Ph- cells in some CML patients in cytogenetic remission to imatinib but restoration of polyclonal hematopoiesis in the majority. Blood. 2003;101:1941–9. 12411298 10.1182/blood-2002-07-2053

