# Supplementary material for: Clonal dynamics of aggressive systemic mastocytosis on avapritinib therapy
Source: Blood Cancer J. 2024 Oct 14;14(1):179. doi: 10.1038/s41408-024-01157-w (PMC11473837; doi:10.1038/s41408-024-01157-w)
Supplement: Supplementary file 1 — Suppl Material [file 41408_2024_1157_MOESM1_ESM.docx]

**Supplementary Materials**

**PATIENTS**

All subjects provided informed consent to the use of their samples (IRB protocol #45880, University of Utah). ASM-AHN patients were treated on the PATHFINDER trial (NCT03580655) and CMML patients were seen at Huntsman Cancer Institute for clinical care. Clinical data including next generation sequencing (NGS) data are provided below and Suppl. Table 7. Suppl.Fig.8 shows key hematologic parameters and dose intensity during treatment. Suppl.Fig.9 shows bone marrow histology prior to and on avapritinib treatment.

**Pt1.** A 58-year-old woman presented with fatigue and abdominal distension, splenomegaly, and ascites. CBC showed monocytosis, neutrophilia, and leukoerythroblastosis. BM biopsy (BMB) revealed multifocal dense aggregates of spindle shaped MCs. BM karyotype was normal. She was started on midostaurin but continued to require frequent paracenteses and was switched to avapritnib 200 mg daily, with reduction in ascites volume and reduction of tryptase (Suppl. Fig. 8 Pt1). BMB on Day 74 (D74) showed reduced MC infiltration, but persistence of AHN (Suppl. Fig. 9A) The patient required avapritinib dose interruption and reduction for Grade 3 thrombocytopenia and a subdural hematoma, but subsequently re-escalated to 200 mg daily. Despite dose reescalation she gradually deteriorated and decided to go on hospice care.

**Pt2.** A 63-year-old woman presented with multifocal sclerotic bone lesions, splenomegaly, retroperitoneal lymphadenopathy, neutropenia, and thrombocytopenia. BMB revealed 50% atypical MCs. Repeat imaging two months later showed progression of splenomegaly, bone lesions, and lymphadenopathy, as well as ascites and pleural effusions requiring chest tube placement. Repeat BMB revealed 70% atypical MCs without dysplasia or increased blasts. Tryptase was 198 ng/mL. She started avapritinib 200 mg daily, with rapid and durable resolution of pleural effusions, and normalization of tryptase. Avapritinib was temporarily paused for pancytopenia, but blood counts stabilized on avapritinib 100 mg daily with G-CSF and eltrombopag support (Suppl. Fig. 8 Pt2). BMB on D56 showed complete resolution of atypical MC infiltrates, but persistent AHN (Suppl. Fig. 9B).

**Pt3.** A 62-year-old woman was diagnosed with telangiectasia eruptiva macularis perstans. Tryptase was 138 ng/mL. Imaging showed extensive mixed sclerotic/lytic bone lesions and mild splenomegaly. BMB was 90% cellular with trilineage hematopoiesis, 10% atypical MC aggregates, and mild dysmegakaryopoiesis. She was referred for clinical trial consideration. Repeat BMB showed SM associated with MDS/MPN-U and Grade 2-3 fibrosis. She started avapritinib 200 mg daily, with a rapid decrease of tryptase. She required dose reduction to 50 mg daily for myelosuppression, with subsequent re-escalation to 100 mg daily with G-CSF support (Suppl. Fig. 8 Pt3). BMB on D1120 showed near-complete resolution of atypical MCs, but persistence of AHN (Suppl. Fig. 9C).

**Pt4.** An asymptomatic 73-year-old woman with a history of allergies was noticed to have eosinophilia (1600/uL, 29% of WBCs). Three months later eosinophils had increased to 3300/uL/41%. BMB demonstrated dysplastic megakaryocytes and 20% atypical MCs. Tryptase was 360 ng/mL. BM cytogenetics and eosinophilia fluorescence in situ hybridization panel were normal. There was no hepatosplenomegaly, and a skeletal survey was normal. She was started on hydroxyurea, with control of eosinophil counts. Two years later, she developed constitutional symptoms associated with increasing leukocytosis and rising eosinophils, myelocytes and metamyelocytes. She started avapritinib 200 mg daily, with rapid decrease of tryptase, normalization of blood counts, and symptom improvement (Suppl. Fig. 8 Pt4). She required dose interruptions and reductions for Grade 3 cytopenias. Two years after starting avapritinib, she presented with leukocytosis and 6% blasts. BMB on D710 was 95% cellular, with 10-15% blasts and 1% scattered atypical MCs (Suppl. Fig. 9D). She was switched to azacytidine plus midostaurin and subsequently cladribine but progressed to AML and passed away.

**METHODS**

**Sample collection, processing, and DNA extraction**

Whole blood was collected prior to starting avapritinib and at several time points on therapy. Whole blood was subjected to red cell lysis with 150 mM NH_4_Cl, 0.1 mM EDTA, and 10 mM KHCO_3_. White blood cells (WBC) were subjected to single cell library preparation within four hours of collection. Skin biopsies obtained during BMB were cultured in collagen-coated 6-well plates (StemCell Technologies) in DMEM supplemented with 20% fetal bovine serum (MilliporeSigma), penicillin/streptomycin (ThermoFisher), and glutamax (ThermoFisher), at 37°C and 5% CO_2_. Upon reaching confluency, cells were trypsinized, moved to a T-175 flask, and harvested when exceeding 80% confluency. DNA was extracted with the Qiagen (Hilden, Germany) DNeasy Blood and Tissue kit and subjected to Qubit for quality control.

**Whole genome sequencing (WGS), data processing and somatic variant calling**

All samples were subjected to 120X WGS using the Illumina NovaSeq. We aligned reads to GRCh37 reference using BWA-MEM (v0.7.17). For each patient, we jointly called single nucleotide variants (SNV) and short insertions/deletions (INDEL) variants from the control skin sample and pre- and post-treatment samples using Freebayes (v1.2.0)^1^. We then filtered the high-quality variants produced by Freebayes by 1) variant quality > 30; 2) per-sample sequencing depth > 30; 3) allele frequency (VAF) in all samples > 0.1; 4) intersecting with 1000G genome accessibility mask; 5) inverse-intersecting with low complexity region mask of GRCh37; 6) filtering out multi-allelic variant sites. Somatic variants were identified when the variant counts were less than 2 and VAF was less than 0.05 in the normal control sample. SNVs and short INDELs were annotated by SnpEff (v4.2)^2^. Somatic copy number variants (CNV) and loss of heterozygosity (LOH) events were called by FACETS, which uses the tumor to normal depth ratio to estimate copy number changes in the tumor and the ratio of alternative allele count to reference allele count (odds ratio) at single nucleotide polymorphism sites in the tumor sample to estimate LOH and allelic imbalance^3^.

**Mutational signature analysis**

We identified mutational signatures using the MutationalPatterns package^4^. Contributions of COSMIC mutational signatures (https://cancer.sanger.ac.uk/signatures/sbs/) to the mutational profile were quantified after *de novo* extraction of mutational signatures from the mutation count matrix.

**Subclone analysis**

We used allele frequencies of somatic variants on copy number 2 heterozygous regions to reconstruct subclone structure and estimate cell prevalence of each subclone as previously described^5-7^. Specifically, we first clustered variants based on their allele frequencies for each sample. Then we used SubcloneSeeker v2 (https://github.com/yiq/SubcloneSeeker/tree/v2) to jointly construct subclone structures^8^.

**Colony Genotyping**

Cryopreserved blood mononuclear cells were thawed and suspended at 10^7^ cells/mL in sorting buffer, blocked with Fc receptor blocking antibody for 10 minutes on ice and then stained with CD34-FITC antibody for 30 mins on ice. Cells were washed twice with 500 μL PBS and stained with 4’,6-diamidino-2-phenylindole (DAPI). CD34^+^ DAPI^-^ cells were sorted on a BD Aria Ill into RPMI, plated at 500 cells/mL in semisolid medium supplemented with recombinant human growth factors (Methocult, Stem Cell Technologies, Cat-# 04230), and cultured in 5% CO_2_ at 37^o^C. At 14 days single colonies were plucked into 100 μL of ALT lysis buffer (Qiagen) and DNA extracted using QIAamp DNA Micro Kit (Qiagen) according to the manufacturer’s instructions. Patient specific variants were amplified with specific primers (Suppl. Table 8) and the amplicons genotyped by Sanger sequencing.

**Single Cell RNA-seq on 10x Genomics Chromium Controller Platform**

Following ammonium chloride-based red cell lysis, white blood cells (WBCs) were resuspended in phosphate buffered saline (PBS) with 0.04% bovine serum albumin, counted and assessed for viability (>70% for all samples). The 10x Genomics Single Cell 3’ Gene Expression Library Prep v3 was used. Following library quality control, the samples were sequenced on NovaSeq 6000 using 30 x 275 bp paired end mode.

**Single cell RNA sequencing (scRNAseq) data processing and analysis**

We used Cellranger (version 3.0.2, 10x Genomics) to align reads to GRCh37 and quantify gene abundance. ScRNAseq from multiple samples were aggregated using the CellRanger command “aggr” with depth normalization. Then we used the Seurat R package^9^ version 3.1.0 for further analysis, including 1) filtering high-quality cells that have less than 5000 genes detected as well as less than 15% mitochondrial transcript counts; 2) normalizing cells with sctransform^10^; 3) visualizing scRNAseq data using uniform manifold approximation and projection (UMAP) plot; 4) clustering cells using nearest neighbor method; 5) identifying cell types using a function, RunAzimuth, in Seurat, as we as known expression markers (Suppl. Table 9). Azimuth, developed as a part of NIH Human Biomolecular Atlas Project (HuBMAP) (<https://azimuth.hubmapconsortium.org/>), is a reference-based automatic tool to annotate cell identity.

**Single cell assignment to subclones**

We developed scBayes, a Bayesian probabilistic framework designed to leverage the subclonal structure derived from bulk DNA sequencing data to identify the subclonal identities of cells from scRNAseq data^11^. In brief, the scBayes algorithm evaluates various hypotheses regarding the identity of a cell, considering tumor subclones or normal cells reconstructed from bulk DNA sequencing data. We used the subclone fractions derived from WGS as prior probabilities for myeloid cells and flat prior probabilities for lymphocytes (Suppl. Table 3-6). scBayes calculates Bayesian posterior probabilities based on positive, negative, or no evidence observed in scRNA-seq data for specific somatic mutations. Finally, scBayes assigns each cell to the subclone with the highest posterior probability. Cells with an assignment quality < 5 or no coverage at any somatic variant site were considered non-informative.

**ADDITIONAL RESULTS and DISCUSSION**

**Mutational landscape of ASM-AHN**

We detected an average of 1891 somatic mutations (Pt1:1259, Pt2:1432, Pt3:1897, Pt4:2977). Three patients had *TET2* mutations (Pt1: L759fs and Y1276fs; Pt2: C834fs and R1134*; Pt3: Q934* and Q755*). In addition, Pt1 had *TP53* R181C and *SRSF2* P95L; Pt2 had *CUX1* P1106fs and heterozygous *CUX1* R672*, which became homozygous due to LOH on chromosome 7. Pt3 had KDM6A splice-site mutation. Pt4 had *ETNK1* N244S, *ASXL1* Y591fs, *EZH2* Y733* and C576Y. While *KIT* D816V was detected by clinical digital droplet PCR in all patients, we did not find *KIT* D816 in Pt1 by WGS. Pt1 had a clonal focal homozygous deletion on chromosome 21 that included *RUNX1*. Pt2 and Pt3 had subclonal LOH on chromosome 7q and chromosome 4q, respectively. Pt4 had a subclonal LOH on chromosome 7q and a subclonal deletion on chromosome 21 (Suppl.Fig.1A-D). We next analyzed mutation signatures according to the criteria of the Pan-Cancer Analysis of Whole Genomes (PCAWG) Consortium of the International Cancer Genome Consortium (ICGC) and The Cancer Genome Atlas (TCGA)^12^. Mutation types were similar across all patients, with dominance of C>T mutations (Suppl.Fig.2A). SBS1 (deamination of 5-methylcytosine) was dominant in pre-avapritinib samples (Suppl.Fig.2B). All samples showed SBS19, SBS89, and SBS5 (Pt4 only post-avapritinib). SBS5 is associated with aging, while the etiology of SBS19 and SBS89 is unknown. Only Pt1 exhibited SBS42, which suggests haloalkane exposure^12^. Mutational signatures were similar in pre- and post-avapritinib samples, except in the post-treatment sample of Pt3, which was enriched for SBS5 (Suppl.Fig.2B).

**CNV analysis**

Pt4 was initially thought to have a chr18 deletion (the ratio of depth in tumor to depth in normal <1). However, further investigation revealed that the “normal” skin sample in this patient had chr18 amplification (Suppl. Fig. 1D and E). This was supported by two lines of evidence. 1) The median depth of germline variants on chr18 for the skin sample is higher than the median depth of germline variants on chr17 and chr19 (189 for chr18 vs. 171 for chr17 and 174 for chr19), whereas the median depth of germline variants on chr18 is similar or lower to chr17 and chr19 in pre and post treatment blood samples (pre: 164 for chr18 vs. 180 for chr17 and 184 for chr19; post: 133 for chr18 vs. 144 for chr17 and 146 for chr19) (Suppl. Fig. 1E). 2) The allele frequencies of the germline variants on chr18 are centered around 0.5 (heterozygous variants) and 1 (homozygous variants) in the two blood samples whereas the allele frequencies of the germline variants on chr18 are centered around 0.4 and 0.6 (allele imbalance) and 1 (homozygous variants) in the skin sample (Suppl. Fig. 1E). Considering the higher depth of chr18, we concluded chr18 was amplified in the skin sample, and the two blood samples have the normal chr18.

**Clonal structure validation by genotyping clones**

To validate the clonal structure, we cultured CD34^+^ cells purified from MNCs cryopreserved prior to and on therapy in cytokine-supplemented semisolid media (available from Pts1, 3 and 4). At two weeks, only pretherapeutic samples grew granulocyte-macrophage colonies. We genotyped ~30 colonies/patient by Sanger sequencing for selected mutations (Suppl.Fig.5). For all three patients, we validated the presence of driver mutations in all colonies. For example, all colonies contained *TET2* and *TP53* mutations in Pt2 and *ETNK1* and *ASXL1* mutations in Pt4. The genotypes of colonies from Pt3 suggested that they all originated from SC5 that contained chromosome 4q22.1-q35.2 LOH, which showed wild type *TET2* and homozygous polymorphism in *ADH1C*. In Pt4, most colonies seemed to originate from subclone SC3 with chromosome 7q21.3-q22.12 LOH, as we observed homozygous *EZH2* Y733* and *MET* D68E due to the LOH event, as well as mutations in *RIT1* and *DPPA4* which were acquired in SC3. Unexpectedly, several colonies had both SC3 and *KIT* mutations, likely reflecting contamination by adjacent cells from another colony. Resequencing was not feasible as no more DNA was available. In both Pt3 and Pt4 a substantial fraction of mutations identified by NGS was not detected in any colony, suggesting that the *in vitro* culture introduced a strong bias, and that analysis of single colonies alone is insufficient to resolve subclone structure.

**Cell population dynamics on avapritinib**

Monocytes from Pt1 and Pt2 initially clustered away from controls but moved closer on avapritinib. This was unexpected, as KIT D816V is low or absent in their blood. A possible explanation is that transcriptional aberrations in these monocytes are secondary to systemic effects of KIT D816V+ cells. Monocytes transcriptomes of Pt3 and Pt4 were similar to healthy controls and remained unchanged on avapritinib, suggesting very few monocytes are from the subclone containing KIT D816V, which is consistent with single cell subclone assignment result (Fig.2B, Suppl. Table 5 and 6).

**REFERENCES**

1. Garrison EM, G. Haplotype-based variant detection from short-read sequencing. arXiv [q-bioGN] <http://arxivorg/abs/12073907>; 2012.

2. Cingolani P, Platts A, Wang le L, et al. A program for annotating and predicting the effects of single nucleotide polymorphisms, SnpEff: SNPs in the genome of Drosophila melanogaster strain w1118; iso-2; iso-3. *Fly (Austin)*. 2012;6(2):80-92.

3. Shen R, Seshan VE. FACETS: allele-specific copy number and clonal heterogeneity analysis tool for high-throughput DNA sequencing. *Nucleic Acids Res*. 2016;44(16):e131.

4. Blokzijl F, Janssen R, van Boxtel R, Cuppen E. MutationalPatterns: comprehensive genome-wide analysis of mutational processes. *Genome Med*. 2018;10(1):33.

5. Black GS, Huang X, Qiao Y, et al. Subclonal evolution of CLL driver mutations is associated with relapse in ibrutinib- and acalabrutinib-treated patients. *Blood*. 2022;140(4):401-405.

6. Than H, Qiao Y, Huang X, et al. Ongoing clonal evolution in chronic myelomonocytic leukemia on hypomethylating agents: a computational perspective. *Leukemia*. 2018;32(9):2049-2054.

7. Huang X, Qiao Y, Brady SW, et al. Novel temporal and spatial patterns of metastatic colonization from breast cancer rapid-autopsy tumor biopsies. *Genome Med*. 2021;13(1):170.

8. Qiao Y, Quinlan AR, Jazaeri AA, Verhaak RG, Wheeler DA, Marth GT. SubcloneSeeker: a computational framework for reconstructing tumor clone structure for cancer variant interpretation and prioritization. *Genome Biol*. 2014;15(8):443.

9. Stuart T, Butler A, Hoffman P, et al. Comprehensive Integration of Single-Cell Data. *Cell*. 2019;177(7):1888-1902.e1821.

10. Hafemeister C, Satija R. Normalization and variance stabilization of single-cell RNA-seq data using regularized negative binomial regression. *Genome Biol*. 2019;20(1):296.

11. Qiao Y, Huang X, Moos PJ, et al. A Bayesian framework to study tumor subclone-specific expression by combining bulk DNA and single-cell RNA sequencing data. *Genome Res*. 2024.

12. Alexandrov LB, Kim J, Haradhvala NJ, et al. The repertoire of mutational signatures in human cancer. *Nature*. 2020;578(7793):94-101.
