# Supplementary material for: Clonal dynamics of aggressive systemic mastocytosis on avapritinib therapy
Source: Blood Cancer J. 2024 Oct 14;14(1):179. doi: 10.1038/s41408-024-01157-w (PMC11473837; doi:10.1038/s41408-024-01157-w)
Supplement: Supplementary file 2 — Suppl Figures [file 41408_2024_1157_MOESM2_ESM.pdf]

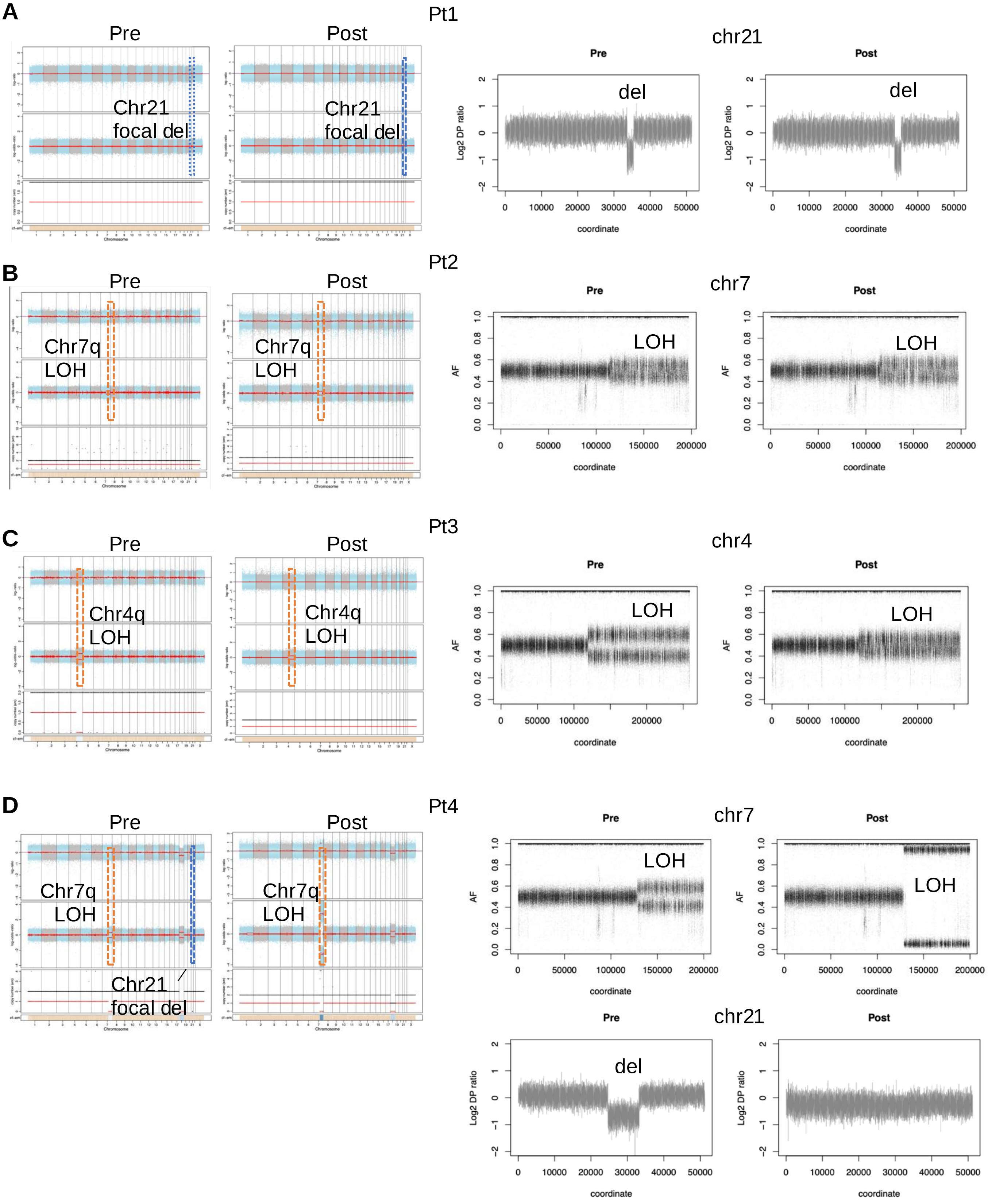

Supplementary Figure 1

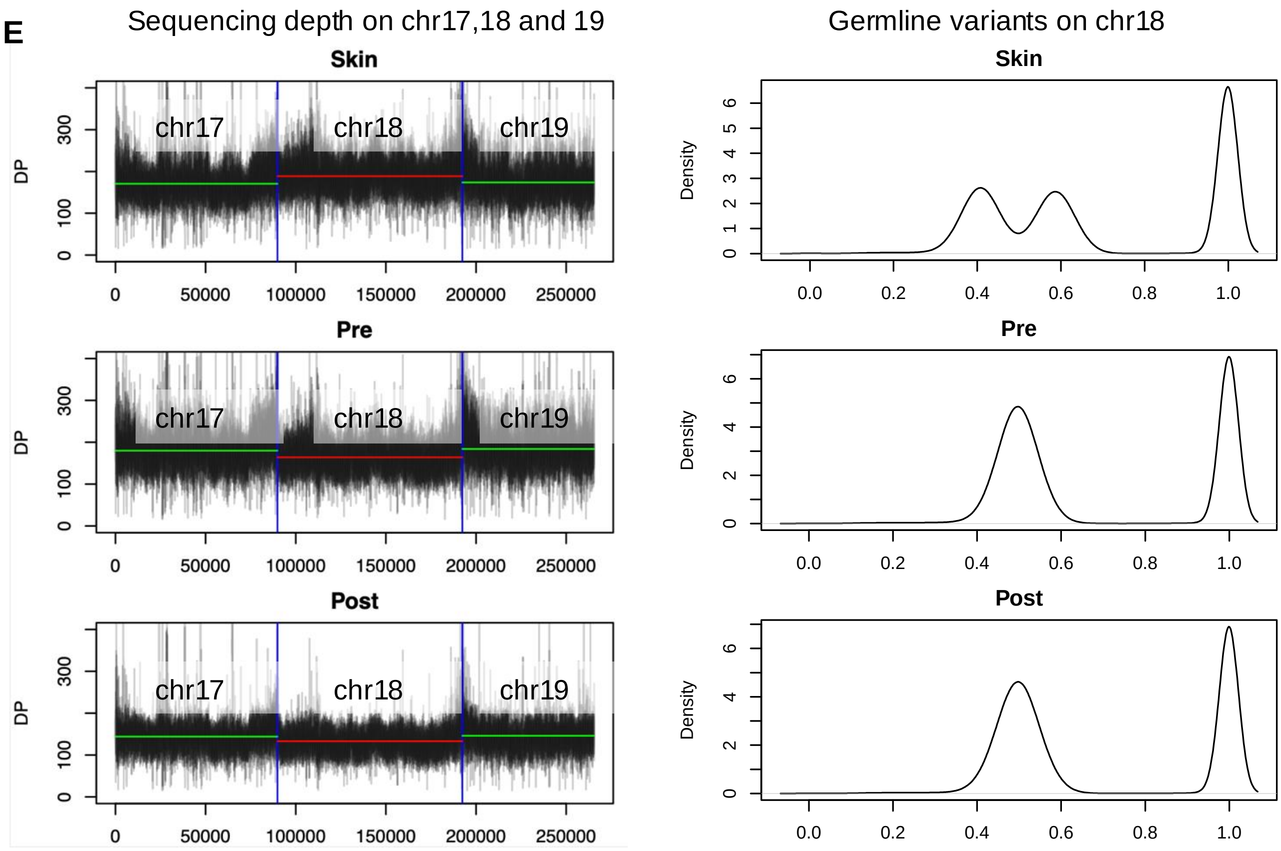

Supplementary Figure 1. Copy number profiles of pre- and post- avapritinib treatment in ASM-AHN patients. (A-D, left panel) Global view of copy number profile. (A-D, right panel) zoom in view of the copy number event for (A) - (D). (A) Pt1: Focal deletion in chromosome 21 spanning the *RUNX1* locus in both pre- and post- treatment samples. (B) Pt2: Subclonal chromosome 7 LOH in both pre- and post- treatment samples. (C) Pt3: Subclonal chromosome 4 LOH in both pre- and post- treatment samples. (D) Pt4: Subclonal chromosome 7q LOH and subclonal chromosome 21 deletion spanning the *RUNX1* locus in the pre-treatment sample, which was undetectable in the post-treatment sample. (E) Pt4: skin sample: Subclonal chromosome 18 amplification. White blood cells had normal copy number on chromosome 18. The left panel is the sequencing depth of chr17, chr18 and chr19 for the skin, pre- and post- avapritinib blood samples. The skin sample showed elevated sequencing depth of chr18 compared to chr17 and chr19. The right panel showed the distribution of the germline variant frequencies in skin (top), pre (middle) and post (bottom) samples. When having no copy number event, there should be 1 peak at the 0.5 for the germline mutation as in the pre and post sample. Two peaks near 0.5 in the skin sample indicates a copy number event.

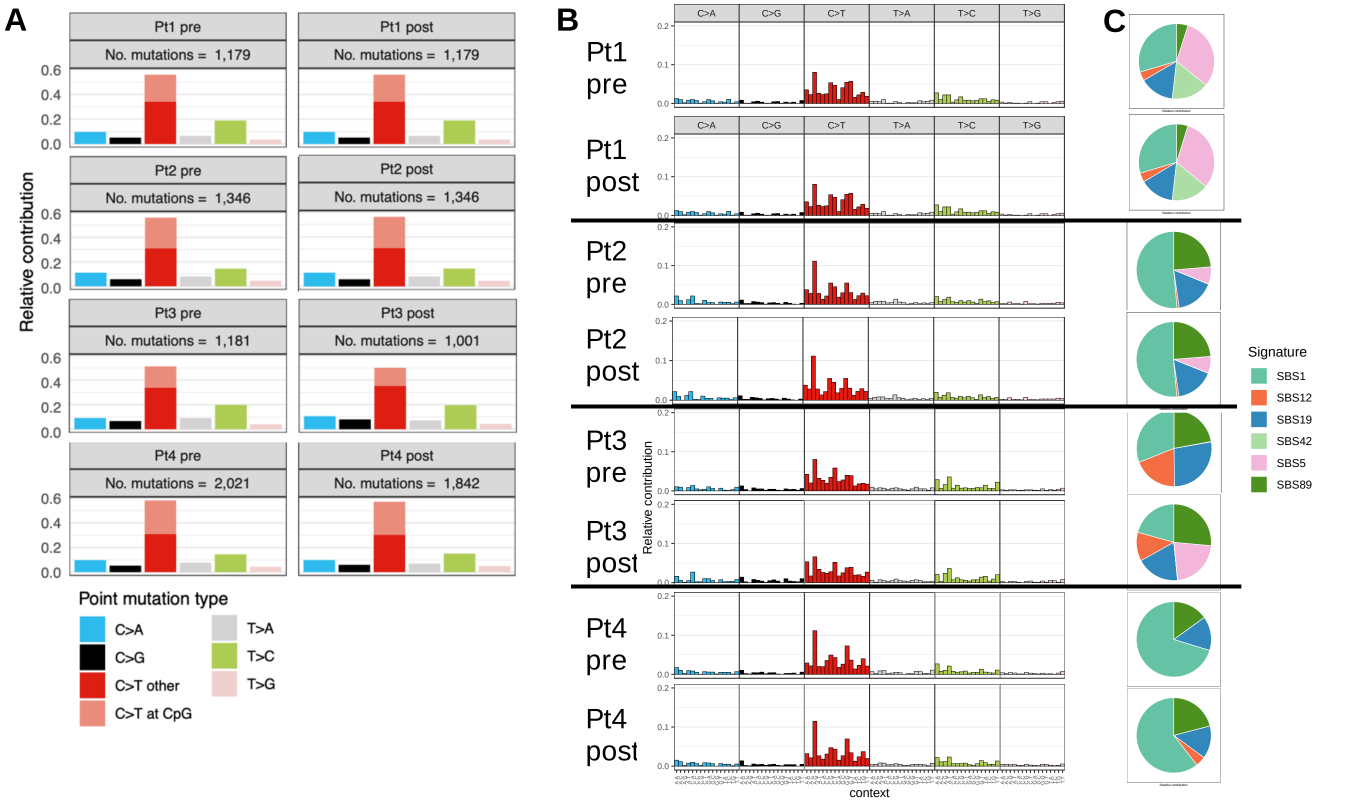

Supplementary Figure 2. Mutational signatures in pre- and post-avapritinib samples in ASM-AHN patients. (A) Point mutation types. (B) Mutation types and context. (C) Mutation signatures.

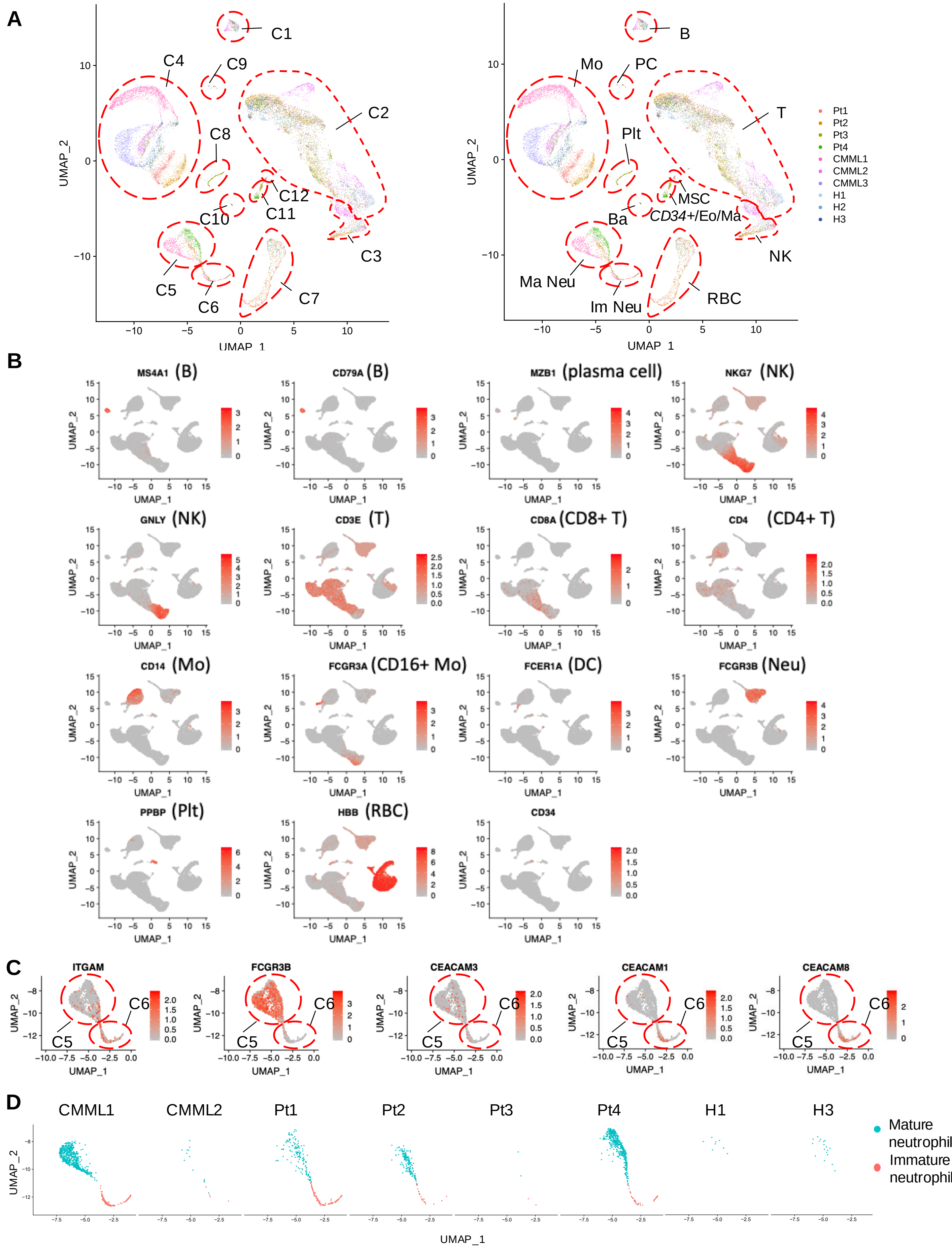

Supplementary Figure 3

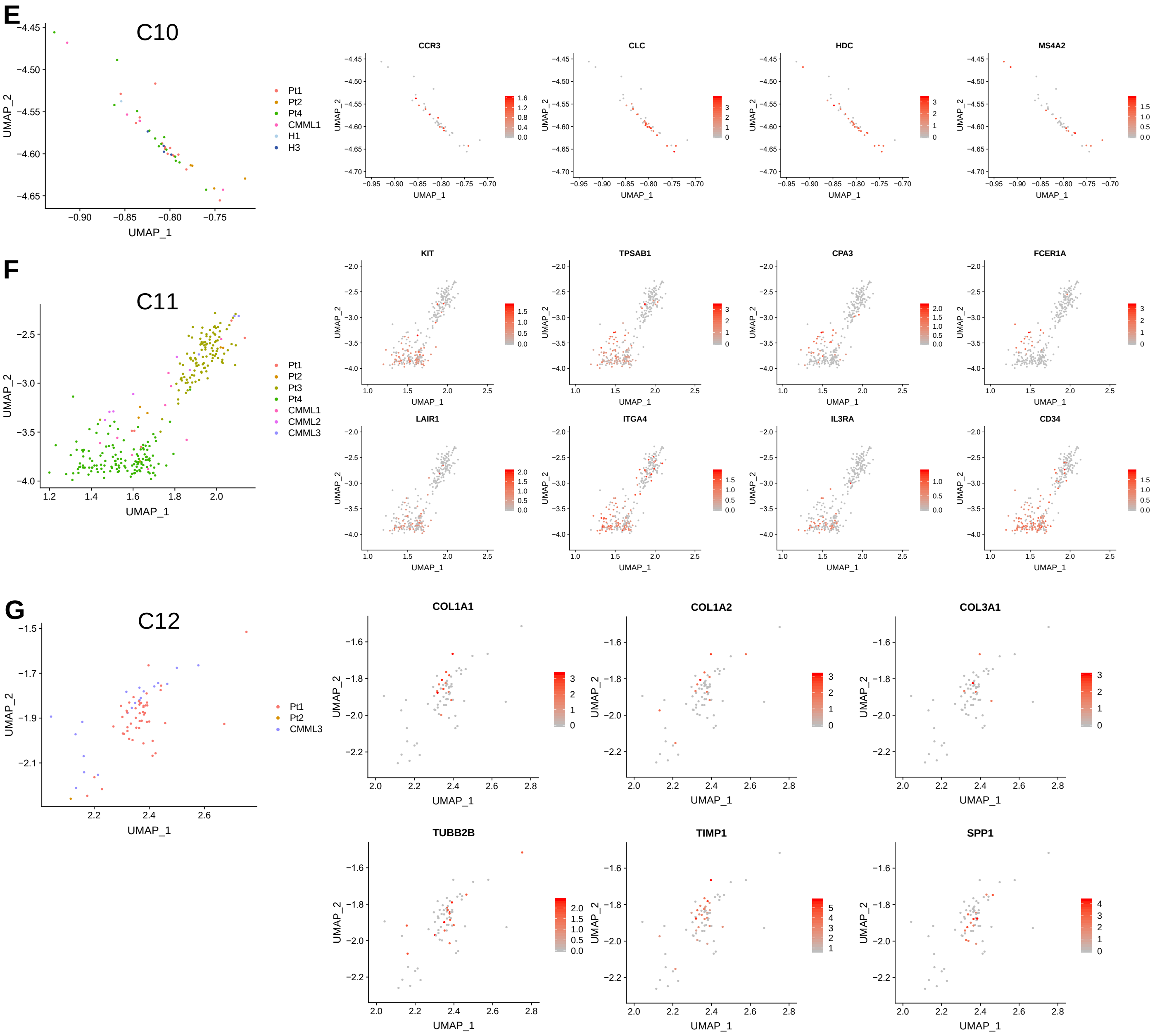

Supplementary Figure 3. Single cell RNA sequencing of white blood cells from therapy-naïve ASM-AHN patients. White blood cells from ASM-AHN patients, treatment-naïve CMML patients, and age-matched healthy controls were subjected to single cell RNA sequencing. (A) Aggregated UMAP plot of all cells with cell clusters (left) and assignment of clusters to cell types (right). (B) Expression of lineage-defining hematopoietic cell markers. (C) Cluster 5 cells express mature neutrophil markers ITGAM, CEACAM3, FCGR3B, whereas cluster 6 expresses immature neutrophil markers CEACAM1, CEACAM8. (D) Immature neutrophils are consistently detected in CMML and ASM-AHN patients, but not in healthy donors. (E) C10 cells express genes (CCR3, CLC, HDC, MS4A2) associated with basophil differentiation. (F) C11 cells express CD34, as well as genes associated with mature eosinophils (LAIR1, ITGA4, IL3RA) and mast cells (KIT, TPSAB1, CPA3). (G) C12 cells express collagen markers such as COL1A1, COL1A2 and COL3A1, and inflammatory marker *TIMP1* and osteopontin (*SPP1*). B – B cells; Ba – basophils; DC – dendritic cells; CD34+/Eo/Ma – cells express CD34 and eosinophils and mast cells markers; Im Neu – immature neutrophils; Ma Neu – mature neutrophils; Mo – monocytes; MSC – mesenchymal stromal cells; NK – natural killer cells; RBC – red blood cells; PC – plasma cells; Plt – platelets; T – T cells.

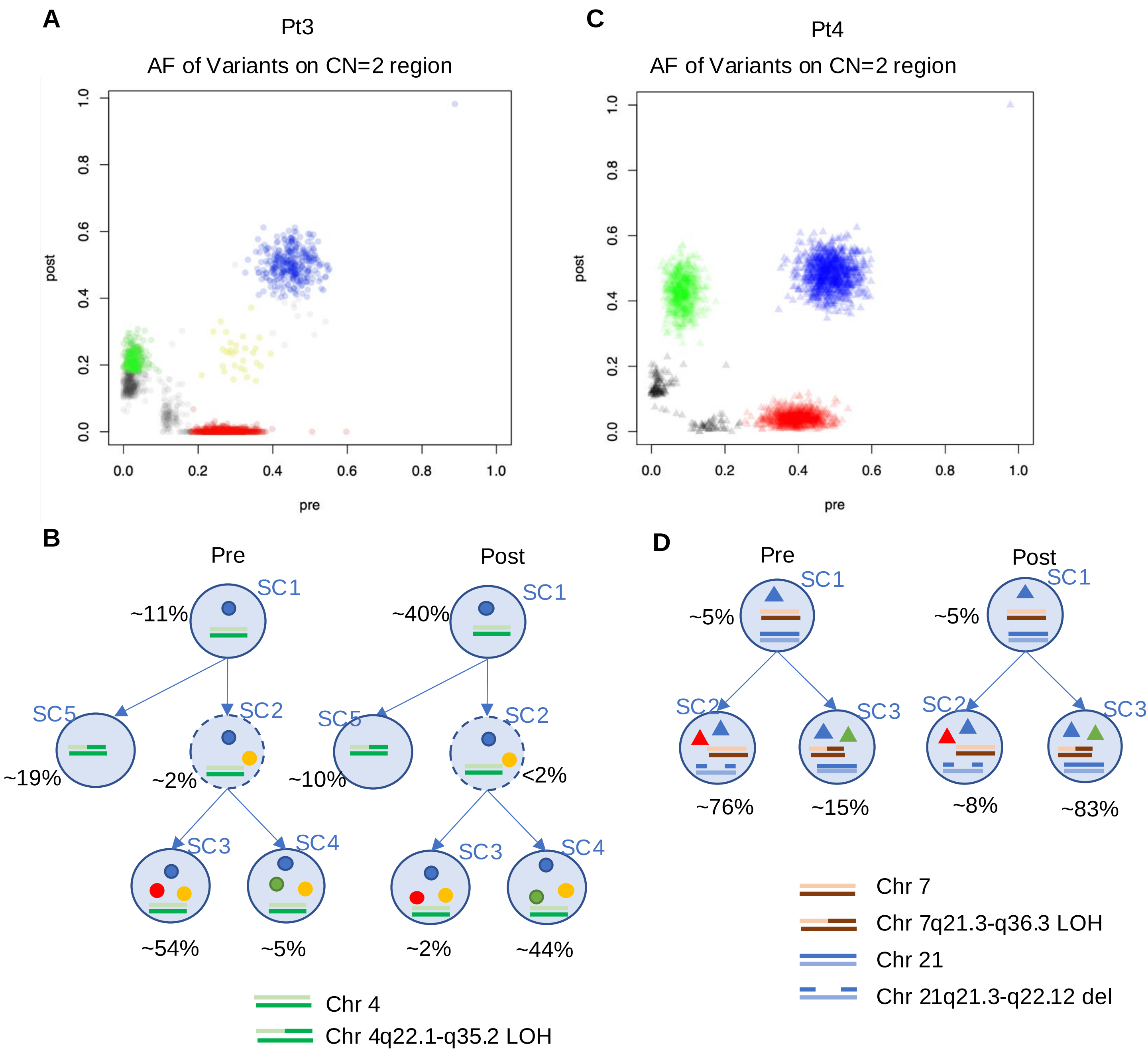

Supplementary Figure 4. Clonal dynamics on avapritinib therapy in Pt3 and Pt4. (A) Variant allele frequencies of somatic mutations in the copy number neutral region in pre- and post-treatment samples from Pt3. Each dot presents a variant. The variants were colored by clusters. (B) Subclone structure of pre- and post- treatment samples in Pt3. Each circle represents a subclone. The subclone identities are labeled beside each circle in blue (SC1-SC5). The dots in the circles represent the clusters of variants. Colors correspond to the variants shown in (A). Arrows represent the parent – offspring relationship between subclones. For example, the subclone containing only the blue dot (variants) gave rise to the subclone that contains both the blue and yellow dots. The cell fraction of each subclone is labeled as percentage next to the subclone. The copy number events such as chromosome 4q22.1-q35.2 LOH was included in the subclone structure. (C) Variant allele frequencies of somatic mutations in the copy number neutral region in pre- and post-treatment samples from Pt4. (D) Subclone structure of pre- and post-treatment samples in Pt4. The subclone identities are labeled beside each circle in blue (SC1-SC3). The copy number events such as chromosome 7q21.3-q36.3 LOH and chromosome 21q21.3-q22.12 deletion were included in the subclone structure.

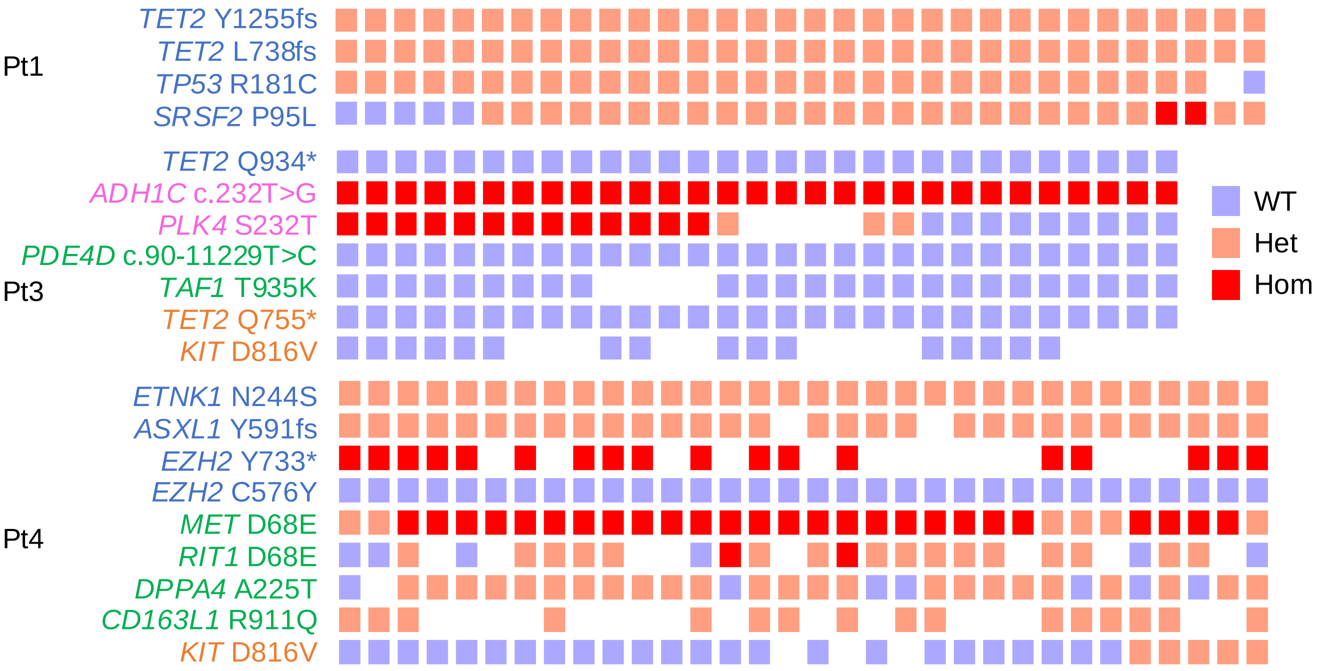

Supplementary Figure 5. Genotyping the colonies grown from CD34+ cells. Each row represents a variant, and each column represents a single colony. Blue color indicates wild type, orange color indicates heterozygous mutation and red color indicates homozygous mutation. The fond color of the variants corresponds to the color used in the fish plots shown in Fig. 2B. Ambiguous sequencing results were excluded (white space).

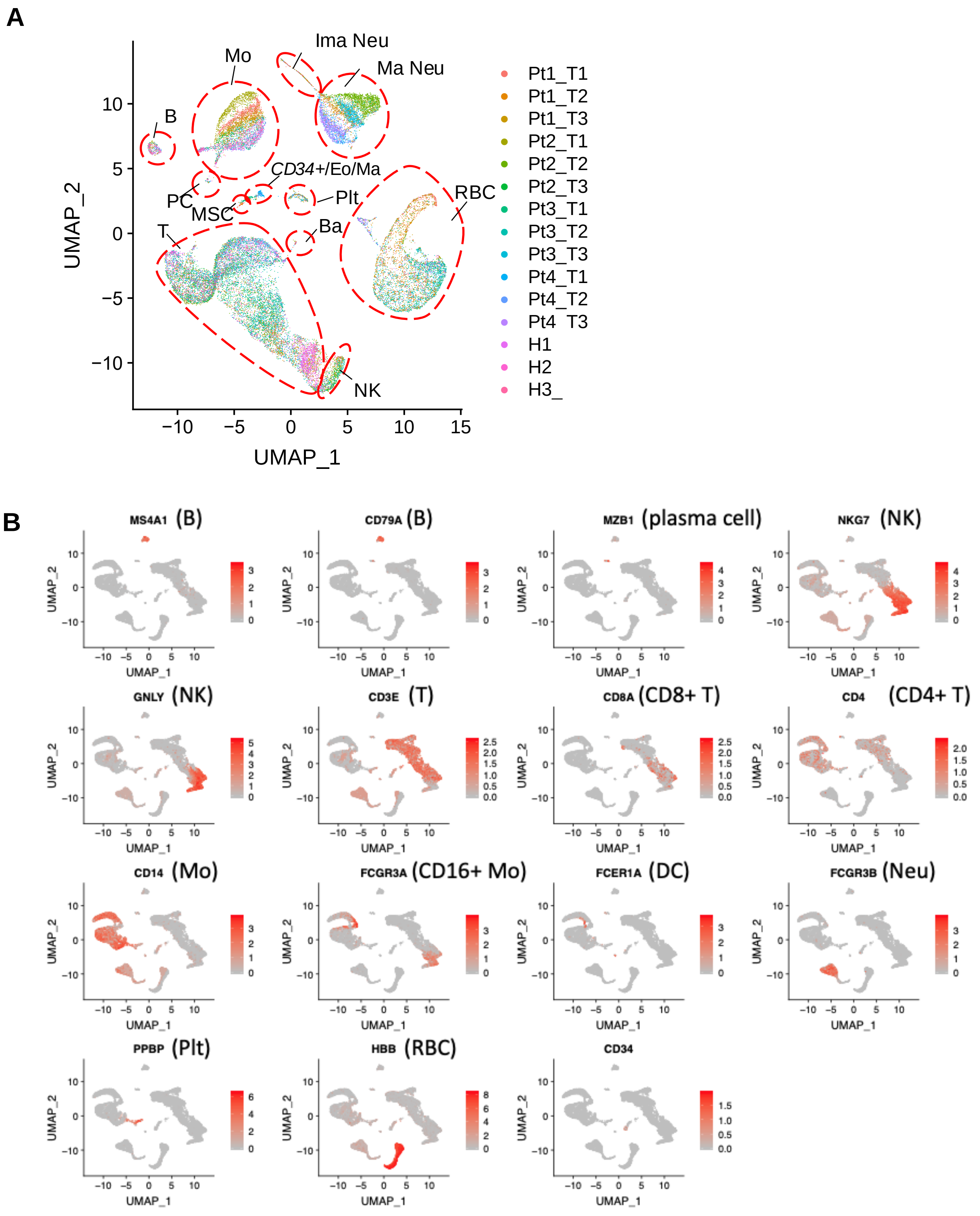

Supplementary Figure 6. Longitudinal single cell RNA sequencing of white blood cells from 4 ASM-AHN patients. (A) Aggregated scRNAseq data from white blood cells from ASM-AHN Pts1-4 at three timepoints, two of which were after initiating treatment. B – B cells; Ba – basophils; DC – dendritic cells; CD34+/Eo/Ma – cells express CD34 and eosinophils and mast cells markers; Im Neu – immature neutrophils; Ma Neu – mature neutrophils; Mo – monocytes; MSC – mesenchymal stromal cells; NK – natural killer cells; RBC – red blood cells; PC – plasma cells; Plt – platelets; T – T cells. (B) Expression of lineage-defining hematopoietic cell markers.

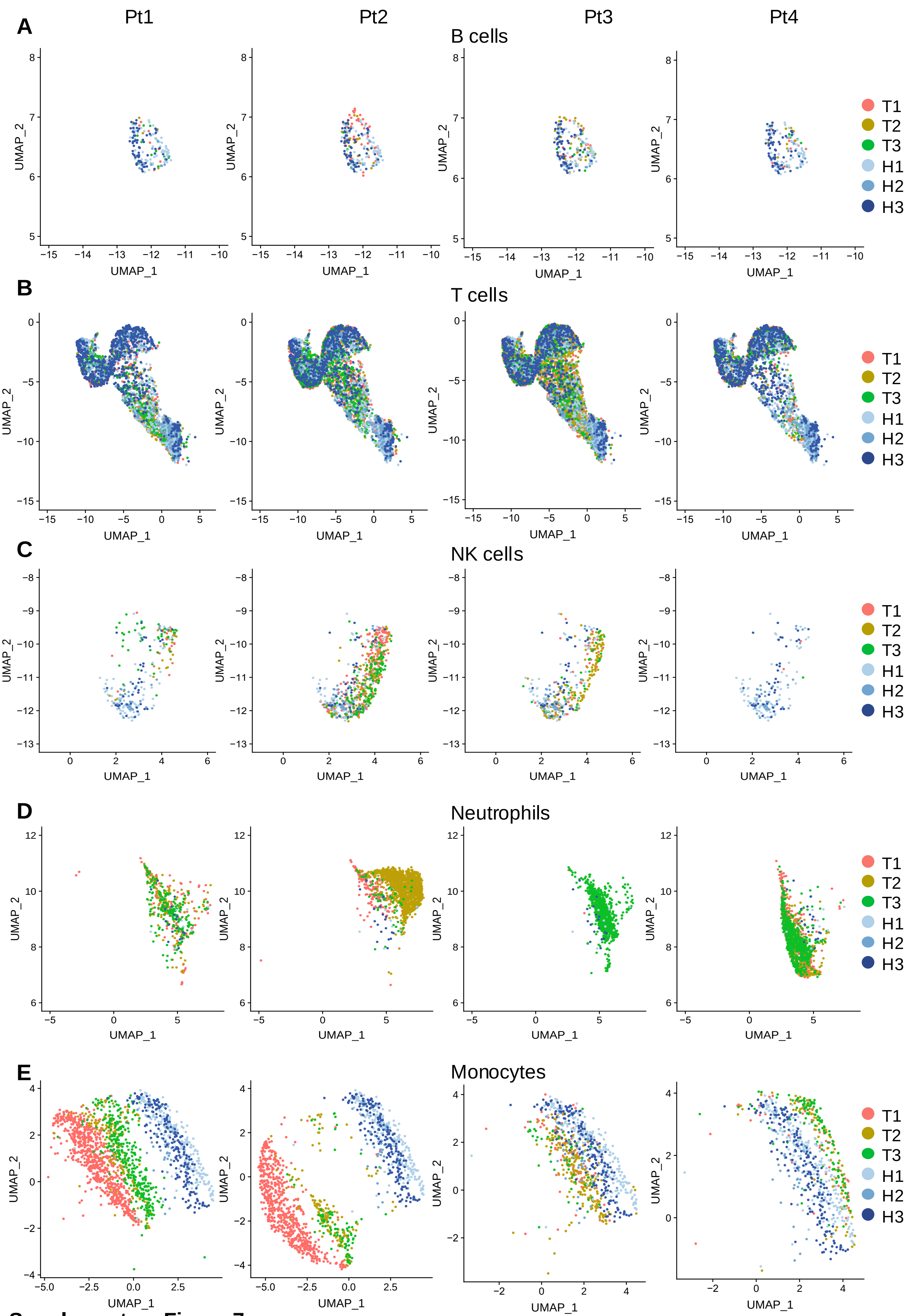

Supplementary Figure 7.

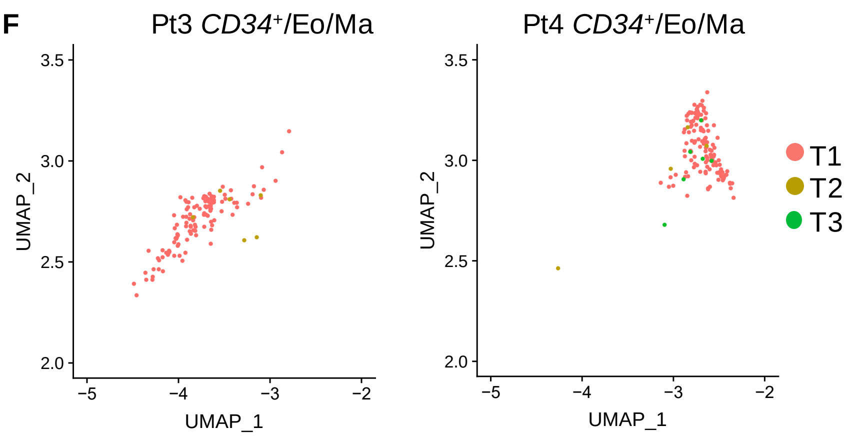

CD34⁺/Eo/Ma	% of WBC
Pt3 T1	5.6%
Pt3 T2	0.2%
Pt3 T3	<0.1%
Pt4 T1	11%
Pt4 T2	0.5%
Pt4 T3	0.4%

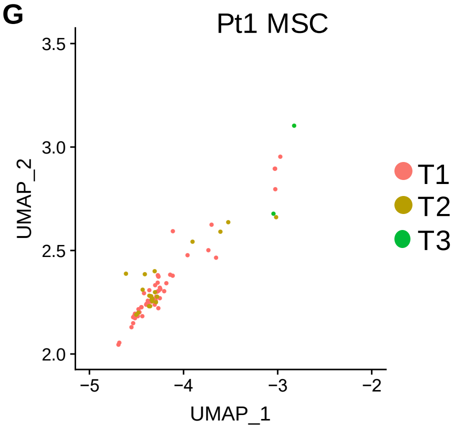

Putative MSC	% of WBC
Pt1 T1	3.1%
Pt1 T2	2.3%
Pt1 T3	<0.1%

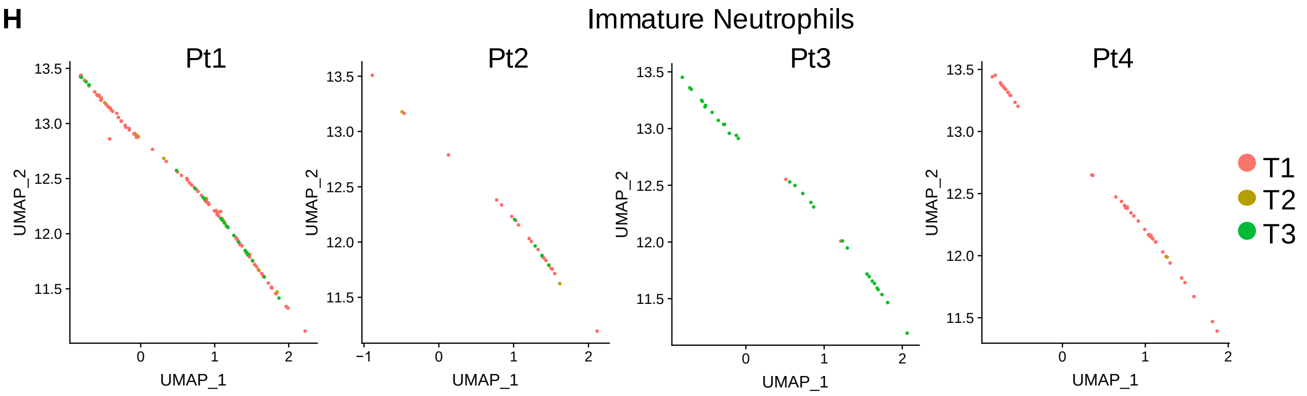

Ima neu	% of WBC
Pt1 T1	6%
Pt1 T2	1%
Pt1 T3	1.6%

Ima neu	% of WBC
Pt2 T1	0.8%
Pt2 T2	<0.1%
Pt2 T3	0.2%

Ima neu	% of WBC
Pt3 T1	<0.1%
Pt3 T2	<0.1%
Pt3 T3	1.7%

Ima neu	% of WBC
Pt4 T1	3.5%
Pt4 T2	0.1%
Pt4 T3	<0.1%

Supplementary Figure 7. Cell population-specific transcriptional dynamics on avapritinib. (A) B cells. (B) T cells. (C) NK cells. (D) Neutrophils. (E) Monocytes. (F) *CD34*⁺/Eo/Ma cell population in Pt3 and Pt4. (G) Collagen gene enriched cell population in Pt1. (H) Immature neutrophils.

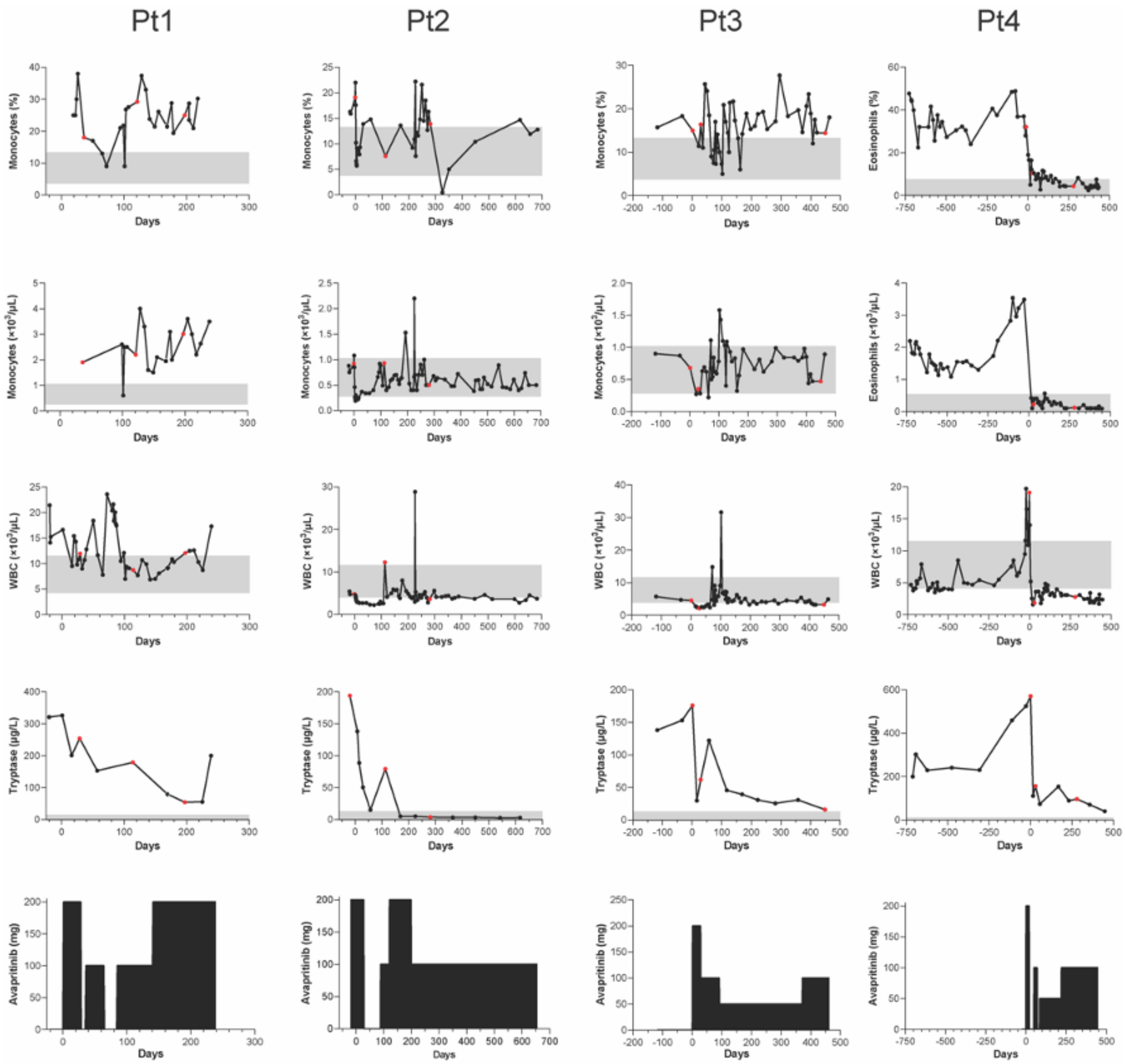

Supplementary Figure 8. Disease activity parameters and avapritinib dose intensity in ASM-AHN patients. Pt1 – Pt4 enrolled in the PATHFINDER study of avapritintib (NCT03580655). Dose adjustments were made according to protocol. Pt.4 initially failed screening for the EXPLORER study of avapritinib (NCT02561988), but subsequently re-screened and enrolled in PATHFINDER. Red dots indicate the time points at which samples were subjected to scRNAseq. WBC – white blood cells.

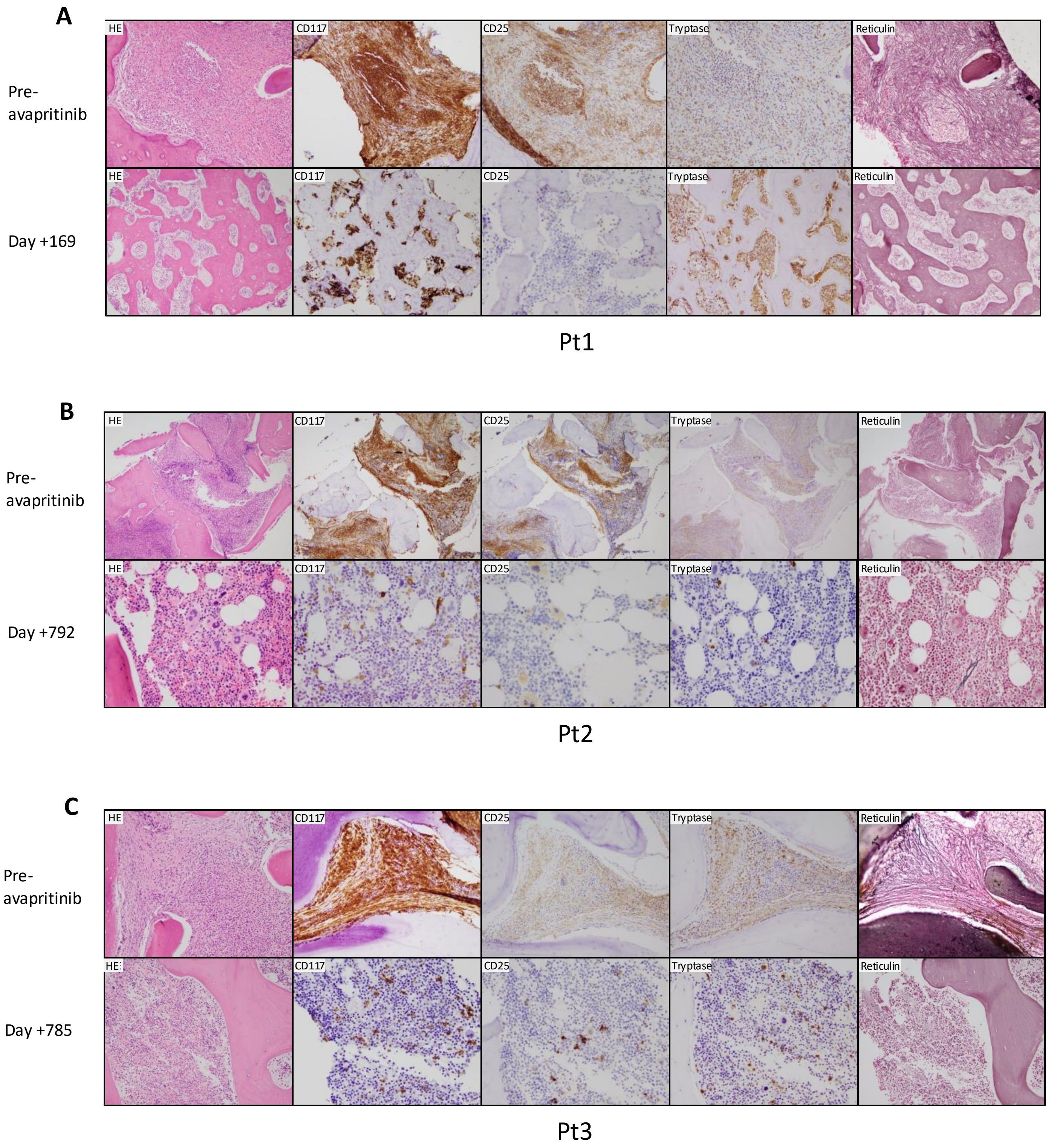

Supplementary Figure 9.

D

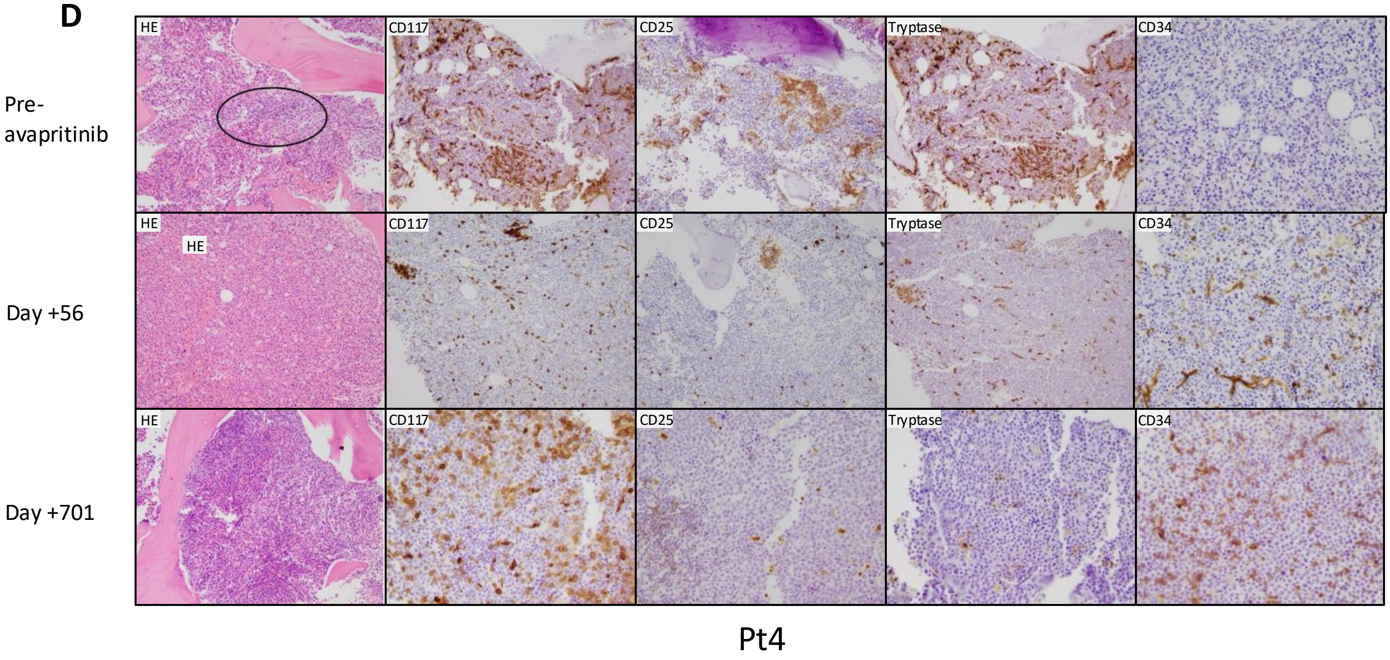

Supplementary Figure 9. Bone marrow histology pre and on avapritinib therapy. (A) Pt1 pre-treatment and on day 169. (B) Pt2 pre-treatment and on day 792. (C) Pt3 pre-treatment and on day 785. (D) Pt4 pre-treatment, on day 56 and day 701.
