# Supplementary material for: Clonal dynamics of aggressive systemic mastocytosis on avapritinib therapy
Source: Blood Cancer J. 2024 Oct 14;14(1):179. doi: 10.1038/s41408-024-01157-w (PMC11473837; doi:10.1038/s41408-024-01157-w)
Supplement: Supplementary file 3 — Suppl Table 1 Cell count per cell type [file 41408_2024_1157_MOESM3_ESM.pdf]

Cell count per cell types

	all cells	nucleated cells	myeloid cells	monocytes	monocytes %nucleated	monocytes %myeloid	mature neutrophils	mature neutrophils %nucleated	mature neutrophils %myeloid	immature neutrophils	immature neutrophils %nucleated	immature neutrophils %m	basophils	basophils %nucleated	basophils %myeloid	C11	C11 %nucleated	C11 %myeloid	C12	C12 %nucleated	C12 %myeloid
Pt1_T1	2058	1594	1053	731	0.459	0.694	144	0.090	0.137	99	0.062	0.094	14	0.009	0.013	5	0.003	0.005	49	0.031	0.047
Pt1_T2	1540	747	353	169	0.226	0.479	137	0.183	0.388	8	0.011	0.023	2	0.003	0.006	4	0.005	0.011	17	0.023	0.048
Pt1_T3	1734	1390	768	339	0.244	0.441	206	0.148	0.268	22	0.016	0.029	2	0.001	0.003	2	0.001	0.003	0	0.000	0.000
Pt2_T1	2784	2309	854	648	0.281	0.759	162	0.070	0.190	20	0.009	0.023	4	0.002	0.005	5	0.002	0.006	1	0.000	0.001
Pt2_T2	2170	2020	1494	173	0.086	0.116	1287	0.637	0.861	2	0.001	0.001	0	0.000	0.000	1	0.000	0.001	0	0.000	0.000
Pt2_T3	1914	1593	229	90	0.056	0.393	63	0.040	0.275	4	0.003	0.017	0	0.000	0.000	5	0.003	0.022	0	0.000	0.000
Pt3_T1	2131	2059	338	99	0.048	0.293	2	0.001	0.006	2	0.001	0.006	1	0.000	0.003	114	0.055	0.337	0	0.000	0.000
Pt3_T2	4055	2715	347	219	0.081	0.631	0	0.000	0.000	0	0.000	0.000	0	0.000	0.000	6	0.002	0.017	0	0.000	0.000
Pt3_T3	2250	1774	958	56	0.032	0.058	858	0.484	0.896	30	0.017	0.031	0	0.000	0.000	0	0.000	0.000	0	0.000	0.000
Pt4_T1	1346	1230	817	51	0.041	0.062	470	0.382	0.575	44	0.036	0.054	14	0.011	0.017	135	0.110	0.165	0	0.000	0.000
Pt4_T2	795	761	409	93	0.122	0.227	259	0.340	0.633	1	0.001	0.002	4	0.005	0.010	4	0.005	0.010	0	0.000	0.000
Pt4_T3	1523	1265	795	113	0.089	0.142	668	0.528	0.840	0	0.000	0.000	2	0.002	0.003	6	0.005	0.008	0	0.000	0.000
H1	1697	1581	351	216	0.137	0.615	8	0.005	0.023	0	0.000	0.000	1	0.001	0.003	0	0.000	0.000	0	0.000	0.000
H2	577	527	91	51	0.097	0.560	0	0.000	0.000	0	0.000	0.000	0	0.000	0.000	0	0.000	0.000	0	0.000	0.000
H3	1234	1195	300	193	0.162	0.643	18	0.015	0.060	0	0.000	0.000	4	0.003	0.013	0	0.000	0.000	0	0.000	0.000
