# Supplementary material for: Clonal dynamics of aggressive systemic mastocytosis on avapritinib therapy
Source: Blood Cancer J. 2024 Oct 14;14(1):179. doi: 10.1038/s41408-024-01157-w (PMC11473837; doi:10.1038/s41408-024-01157-w)
Supplement: Supplementary file 4 — Suppl Table 2 Informative, non-informative and proportion of clonally attributable cells by cell type [file 41408_2024_1157_MOESM4_ESM.pdf]

Informative, non-informative and proportion of clonally attributable cells by cell type

		Pt1	Pt2	Pt3	Pt4	Total/average %
Myeloid	Total number of cells	2174	2577	1643	2021	8415
	Total number of informative cells	448	503	144	343	1438
	Informative cells (%)	21%	20%	9%	17%	17%
	Informative cells attributable to subclones (%)	100%	100%	100%	99%	100%
	Informative cells non-attributable to subclone (%)	0%	0%	0%	1%	0%
Monocytes	Total number of cells	1239	911	374	257	2781
	Total number of informative cells	410	349	89	89	937
	Informative cells (%)	33%	38%	24%	35%	34%
	Informative cells attributable to subclones (%)	100%	100%	100%	100%	100%
	Informative cells non-attributable to subclone (%)	0%	0%	0%	0%	0%
Neutrophils	Total number of cells	616	1538	892	1442	4488
	Total number of informative cells	29	135	35	158	357
	Informative cells (%)	5%	9%	4%	11%	8%
	Informative cells attributable to subclones (%)	100%	100%	100%	100%	100%
	Informative cells non-attributable to subclone (%)	0%	0%	0%	0%	0%
$CD34^+$ Eo/MC	Total number of cells	11	11	121	145	288
	Total number of informative cells	7	2	2	66	77
	Informative cells (%)	64%	18%	2%	46%	27%
	Informative cells attributable to subclones (%)	100%	100%	100%	97%	99%
	Informative cells non-attributable to subclone (%)	0%	0%	0%	3%	1%
Basophils	Total number of cells	18	4	1	20	43
	Total number of informative cells	2	1	0	8	11
	Informative cells (%)	11%	25%	0%	40%	26%
	Informative cells attributable to subclones (%)	100%	100%	NA	88%	96%
	Informative cells non-attributable to subclone (%)	0	0	NA	12%	4%
Lymphocytes	Total number of cells	1557	3345	4905	1235	11042
	Total number of informative cells	563	1473	2166	577	4779
	Informative cells (%)	36%	44%	44%	47%	43%
	Informative cells attributable to subclones (%)	4%	10%	4%	2%	5%
	Informative cells non-attributable to subclone (%)	96%	90%	96%	98%	95%
B cells	Total number of cells	23	73	70	31	197

	Total number of informative cells	11	33	29	20	93
	Informative cells (%)	48%	45%	41%	65%	47%
	Informative cells attributable to subclones (%)	0%	9%	3%	0%	4%
	Informative cells non-attributable to subclone (%)	100%	91%	97%	100%	96%
T cells	Total number of cells	1434	2716	4582	1195	9927
	Total number of informative cells	523	1188	2015	555	4281
	Informative cells (%)	36%	44%	44%	46%	43%
	Informative cells attributable to subclones (%)	2%	2%	2%	2%	2%
	Informative cells non-attributable to subclone (%)	98%	98%	98%	98%	98%
NK cells	Total number of cells	92	544	243	3	882
	Total number of informative cells	25	243	118	2	388
	Informative cells (%)	27%	45%	49%	67%	44%
	Informative cells attributable to subclones (%)	40%	51%	50%	50%	48%
	Informative cells non-attributable to subclone (%)	60%	49%	50%	50%	52%
