# Supplementary material for: Clonal dynamics of aggressive systemic mastocytosis on avapritinib therapy
Source: Blood Cancer J. 2024 Oct 14;14(1):179. doi: 10.1038/s41408-024-01157-w (PMC11473837; doi:10.1038/s41408-024-01157-w)
Supplement: Supplementary file 5 — Suppl Table 3 scBayes assignment for Pt1 [file 41408_2024_1157_MOESM5_ESM.pdf]

scBayes assignment for Pt1

Myeloid cells	T1	T2	T3	T1-T3 combined
ASM-AHN	277	51	120	448
Non-clonal	0	0	0	0
Non-informative	776	302	648	1726
Total number of cells	1053	353	768	2174
Total number of informative cells	277	51	120	448
Percentage of informative cells	26%	14%	16%	21%
Percentage of informative cells attributable to subclones	100%	100%	100%	100%

Cell prevalence prior	T1	T2	T3
ASM-AHN	96%	85%	85%
Normal	4%	15%	15%

Monocytes	T1	T2	T3	T1-T3 combined
ASM-AHN	252	47	111	410
Non-clonal	0	0	0	0
Non-informative	479	122	228	829
Total number of cells	731	169	339	1239
Total number of informative cells	252	47	111	410
Percentage of informative cells	34%	28%	33%	33%
Percentage of informative cells attributable to subclones	100%	100%	100%	100%

Neutrophils	T1	T2	T3	T1-T3 combined
ASM-AHN	18	4	7	29
Non-clonal	0	0	0	0
Non-informative	225	141	221	587
Total number of cells	243	145	228	616
Total number of informative cells	18	4	7	29
Percentage of informative cells	7%	3%	3%	5%
Percentage of informative cells attributable to subclones	100%	100%	100%	100%

Basophil	T1	T2	T3	T1-T3 combined
ASM-AHN	2	0	0	2
Non-clonal	0	0	0	0
Non-informative	12	2	2	16
Total number of cells	14	2	2	18
Total number of informative cells	2	0	0	2
Percentage of informative cells	14%	0%	0%	11%
Percentage of informative cells attributable to subclones	100%	na	na	100%

CD34+	T1	T2	T3	T1-T3 combined
ASM-AHN	4	1	2	7
Non-clonal	0	0	0	0
Non-informative	1	3	0	4
Total number of cells	5	4	2	11
Total number of informative cells	4	1	2	7
Percentage of informative cells	80%	25%	100%	64%
Percentage of informative cells attributable to subclones	100%	100%	100%	100%

lymphocytes	T1	T2	T3	T1-T3 combined
ASM-AHN	10	6	4	20
Non-clonal	191	141	211	543
Non-informative	340	247	407	994
Total number of cells	541	394	622	1557
Total number of informative cells	201	147	215	563
Percentage of informative cells	37%	37%	35%	36%
Percentage of informative cells attributable to subclones	5%	4%	2%	4%

Cell prevalence prior	T1	T2	T3
ASM-AHN	50%	50%	50%
Normal	50%	50%	50%

B	T1	T2	T3	T1-T3 combined
ASM-AHN	0	0	0	0
Non-clonal	2	2	7	11
Non-informative	5	4	3	12
Total number of cells	7	6	10	23
Total number of informative cells	2	2	7	11
Percentage of informative cells	29%	33%	70%	48%
Percentage of informative cells attributable to subclones	0%	0%	0%	0%

T	T1	T2	T3	T1-T3 combined
ASM-AHN	4	3	2	9
Non-clonal	183	133	198	514
Non-informative	319	226	366	911
Total number of cells	506	362	566	1434
Total number of informative cells	187	136	200	523
Percentage of informative cells	37%	38%	35%	36%
Percentage of informative cells attributable to subclones	2%	2%	1%	2%

NK	T1	T2	T3	T1-T3 combined
ASM-AHN	5	3	2	10
Non-clonal	4	6	5	15
Non-informative	15	17	35	67
Total number of cells	24	26	42	92
Total number of informative cells	9	9	7	25
Percentage of informative cells	38%	35%	17%	27%
Percentage of informative cells attributable to subclones	56%	33%	29%	40%
