# Supplementary material for: Clonal dynamics of aggressive systemic mastocytosis on avapritinib therapy
Source: Blood Cancer J. 2024 Oct 14;14(1):179. doi: 10.1038/s41408-024-01157-w (PMC11473837; doi:10.1038/s41408-024-01157-w)
Supplement: Supplementary file 6 — Suppl Table 4 scBayes assignment for Pt2 [file 41408_2024_1157_MOESM6_ESM.pdf]

scBayes assignment for Pt2

Myeloid cells	T1	T2	T3	T1-T3 combined
ASM-AHN	269	200	33	502
Non-clonal	0	1	0	1
Non-informative	585	1293	196	2074
Total number of cells	854	1494	229	2577
Total number of informative cells	269	201	33	503
Percentage of informative cells	31%	13%	14%	20%
Percentage of informative cells attributable to subclones	100%	100%	100%	100%

Cell prevalence prior	T1	T2	T3
ASM-AHN	96%	96%	96%
Normal	4%	4%	4%

Monocytes	T1	T2	T3	T1-T3 combined
ASM-AHN	253	67	28	348
Non-clonal	0	1	0	1
Non-informative	395	105	62	562
Total number of cells	648	173	90	911
Total number of informative cells	253	68	28	349
Percentage of informative cells	39%	39%	31%	38%
Percentage of informative cells attributable to subclones	100%	99%	100%	100%

Neutrophils	T1	T2	T3	T1-T3 combined
ASM-AHN	8	125	2	135
Non-clonal	0	0	0	0
Non-informative	174	1164	65	1403
Total number of cells	182	1289	67	1538
Total number of informative cells	8	125	2	135
Percentage of informative cells	4%	10%	3%	9%
Percentage of informative cells attributable to subclones	100%	100%	100%	100%

Basophil	T1	T2	T3	T1-T3 combined
ASM-AHN	1	0	0	1
Non-clonal	0	0	0	0
Non-informative	3	0	0	3
Total number of cells	4	0	0	4
Total number of informative cells	1	0	0	1
Percentage of informative cells	25%	na	na	25%
Percentage of informative cells attributable to subclones	100%	na	na	100%

CD34+	T1	T2	T3	T1-T3 combined
ASM-AHN	1	0	1	2
Non-clonal	0	0	0	0
Non-informative	4	1	4	9
Total number of cells	5	1	5	11
Total number of informative cells	1	0	1	2
Percentage of informative cells	20%	0%	20%	18%
Percentage of informative cells attributable to subclones	100%	na	100%	100%

lymphocytes	T1	T2	T3	T1-T3 combined
ASM-AHN	69	25	57	151
Non-clonal	542	170	610	1322
Non-informative	844	331	697	1872
Total number of cells	1455	526	1364	3345
Total number of informative cells	611	195	667	1473
Percentage of informative cells	42%	37%	49%	44%
Percentage of informative cells attributable to subclones	11%	13%	9%	10%

Cell prevalence prior	T1	T2	T3
ASM-AHN	50%	50%	50%
Normal	50%	50%	50%

B	T1	T2	T3	T1-T3 combined
ASM-AHN	2	1	0	3
Non-clonal	18	8	4	30
Non-informative	24	14	2	40
Total number of cells	44	23	6	73
Total number of informative cells	20	9	4	33
Percentage of informative cells	45%	39%	67%	45%
Percentage of informative cells attributable to subclones	10%	11%	0%	9%

T	T1	T2	T3	T1-T3 combined
ASM-AHN	12	4	5	21
Non-clonal	462	136	569	1167
Non-informative	665	249	614	1528
Total number of cells	1139	389	1188	2716
Total number of informative cells	474	140	574	1188
Percentage of informative cells	42%	36%	48%	44%
Percentage of informative cells attributable to subclones	3%	3%	1%	2%

NK	T1	T2	T3	T1-T3 combined
ASM-AHN	53	20	52	125
Non-clonal	57	25	36	118
Non-informative	153	67	81	301
Total number of cells	263	112	169	544
Total number of informative cells	110	45	88	243
Percentage of informative cells	42%	40%	52%	45%
Percentage of informative cells attributable to subclones	48%	44%	59%	51%
