# Supplementary material for: Clonal dynamics of aggressive systemic mastocytosis on avapritinib therapy
Source: Blood Cancer J. 2024 Oct 14;14(1):179. doi: 10.1038/s41408-024-01157-w (PMC11473837; doi:10.1038/s41408-024-01157-w)
Supplement: Supplementary file 7 — Suppl Table 5 scBayes assignment for Pt3 [file 41408_2024_1157_MOESM7_ESM.pdf]

scBayes assignment for Pt3

Myeloid cells	T1	T2	T3	T1-T3 combined
SC1	0	21	8	29
SC2	0	0	0	0
SC3	9	0	0	9
SC4	7	42	57	106
Non-clonal	0	0	0	0
Non-informative	322	284	893	1499
Total number of cells	338	347	958	1643
Total number of informative cells	16	63	65	144
Percentage of informative cells	5%	18%	7%	9%
Percentage of informative cells attributable to subclones	100%	100%	100%	100%

Cell prevalence prior	T1	T2	T3
SC1	30%	50%	50%
SC2	2%	2%	2%
SC3	54%	2%	2%
SC4	5%	44%	44%
Normal	9%	2%	2%

Monocytes	T1	T2	T3	T1-T3 combined
SC1	0	17	3	20
SC2	0	0	0	0
SC3	8	0	0	8
SC4	6	30	25	61
Non-clonal	0	0	0	0
Non-informative	85	172	28	285
Total number of cells	99	219	56	374
Total number of informative cells	14	47	28	89
Percentage of informative cells	14%	21%	50%	24%
Percentage of informative cells attributable to subclones	100%	100%	100%	100%

Neutrophils	T1	T2	T3	T1-T3 combined
SC1	0	0	4	4
SC2	0	0	0	0
SC3	0	0	0	0
SC4	0	0	31	31
Non-clonal	0	0	0	0
Non-informative	4	0	853	857
Total number of cells	4	0	888	892
Total number of informative cells	0	0	35	35
Percentage of informative cells	0%	na	4%	4%
Percentage of informative cells attributable to subclones	na	na	100%	100%

basophils	T1	T2	T3	T1-T3 combined
SC1	0	0	0	0
SC2	0	0	0	0
SC3	0	0	0	0
SC4	0	0	0	0
Non-clonal	0	0	0	0
Non-informative	1	0	0	1
Total number of cells	1	0	0	1
Total number of informative cells	0	0	0	0
Percentage of informative cells	0%	na	na	0%
Percentage of informative cells attributable to subclones	na	na	na	na

CD34	T1	T2	T3	T1-T3 combined
SC1	0	1	0	1
SC2	0	0	0	0
SC3	0	0	0	0
SC4	1	0	0	1
Non-clonal	0	0	0	0
Non-informative	114	5	0	119
Total number of cells	115	6	0	121
Total number of informative cells	1	1	0	2
Percentage of informative cells	1%	na	na	2%
Percentage of informative cells attributable to subclones	na	na	na	100%

Lymphocytes	T1	T2	T3	T1-T3 combined
SC1	0	0	0	0
SC2	0	0	0	0
SC3	5	17	6	28
SC4	15	41	12	68
Non-clonal	718	939	413	2070
Non-informative	983	1371	385	2739
Total number of cells	1721	2368	816	4905
Total number of informative cells	738	997	431	2166
Percentage of informative cells	43%	42%	53%	44%
Percentage of informative cells attributable to subclones	3%	6%	4%	4%

Cell prevalence prior	T1	T2	T3
SC1	13%	13%	13%
SC2	13%	13%	13%
SC3	13%	13%	13%
SC4	13%	13%	13%
Normal	50%	50%	50%

B	T1	T2	T3	T1-T3 combined
SC1	0	0	0	0
SC2	0	0	0	0
SC3	0	0	0	0
SC4	0	0	1	1
Non-clonal	6	18	4	28
Non-informative	12	21	8	41
Total number of cells	18	39	13	70
Total number of informative cells	6	18	5	29
Percentage of informative cells	33%	46%	38%	41%
Percentage of informative cells attributable to subclones	0%	0%	20%	3%

T	T1	T2	T3	T1-T3 combined
SC1	0	0	0	0
SC2	0	0	0	0
SC3	1	5	1	7
SC4	8	18	3	29
Non-clonal	699	887	393	1979
Non-informative	936	1271	360	2567
Total number of cells	1644	2181	757	4582
Total number of informative cells	708	910	397	2015
Percentage of informative cells	43%	42%	52%	44%
Percentage of informative cells attributable to subclones	1%	3%	1%	2%

NK	T1	T2	T3	T1-T3 combined
SC1	0	0	0	0
SC2	0	0	0	0
SC3	4	12	5	21
SC4	7	23	8	38
Non-clonal	12	32	15	59
Non-informative	31	77	17	125
Total number of cells	54	144	45	243
Total number of informative cells	23	67	28	118
Percentage of informative cells	43%	47%	62%	49%
Percentage of informative cells attributable to subclones	48%	52%	46%	50%
