# Supplementary material for: Clonal dynamics of aggressive systemic mastocytosis on avapritinib therapy
Source: Blood Cancer J. 2024 Oct 14;14(1):179. doi: 10.1038/s41408-024-01157-w (PMC11473837; doi:10.1038/s41408-024-01157-w)
Supplement: Supplementary file 8 — Suppl Table 6 scBayes assignment for Pt4 [file 41408_2024_1157_MOESM8_ESM.pdf]

scBayes assignment for Pt4

Myeloid cells	T1	T2	T3	T1-T3 combined
SC1	0	0	0	0
SC2	126	16	20	162
SC3	18	66	94	178
Non-clonal	3	0	0	3
Non-informative	670	327	681	1678
Total number of cells	817	409	795	2021
Total number of informative cells	147	82	114	343
Percentage of informative cells	18%	20%	14%	17%
Percentage of informative cells attributable to subclones	98%	100%	100%	99%

Cell prevalence prior	T1	T2	T3
SC1	5%	5%	5%
SC2	76%	8%	8%
SC3	15%	83%	83%
Normal	4%	4%	4%

CD34+/Im eosinophils/Mast cells	T1	T2	T3	T1-T3 combined
SC1	0	0	0	0
SC2	53	0	0	53
SC3	8	0	3	11
Non-clonal	2	0	0	2
Non-informative	72	4	3	79
Total number of cells	135	4	6	145
Total number of informative cells	63	0	3	66
Percentage of informative cells	47%	0%	50%	46%
Percentage of informative cells attributable to subclones	97%	na	100%	97%

Monocytes	T1	T2	T3	T1-T3 combined
SC1	0	0	0	0
SC2	1	1	1	3
SC3	6	38	42	86
Non-clonal	0	0	0	0
Non-informative	44	54	70	168
Total number of cells	51	93	113	257
Total number of informative cells	7	39	43	89
Percentage of informative cells	14%	42%	38%	35%
Percentage of informative cells attributable to subclones	100%	100%	100%	100%

Neutrophils	T1	T2	T3	T1-T3 combined
SC1	0	0	0	0
SC2	62	14	19	95
SC3	1	16	46	63
Non-clonal	0	0	0	0
Non-informative	451	230	603	1284
Total number of cells	514	260	668	1442
Total number of informative cells	63	30	65	158
Percentage of informative cells	12%	12%	10%	11%
Percentage of informative cells attributable to subclones	100%	100%	100%	100%

Basophil	T1	T2	T3	T1-T3 combined
SC1	0	0	0	0
SC2	5	1	0	6
SC3	0	0	1	1
Non-clonal	1	0	0	1
Non-informative	8	3	1	12
Total number of cells	14	4	2	20
Total number of informative cells	6	1	1	8
Percentage of informative cells	43%	25%	50%	40%
Percentage of informative cells attributable to subclones	83%	100%	100%	88%

Lymphocytes	T1	T2	T3	T1-T3 combined
SC1	0	0	1	1
SC2	4	1	1	6
SC3	1	1	2	4
Non-clonal	202	173	191	566
Non-informative	202	175	275	652
Total number of cells	409	350	470	1229
Total number of informative cells	207	175	195	577
Percentage of informative cells	51%	50%	41%	47%
Percentage of informative cells attributable to subclones	2%	1%	2%	2%

Cell prevalence prior	T1	T2	T3
SC1	16%	16%	16%
SC2	16%	16%	16%
SC3	16%	16%	16%
Normal	50%	50%	50%

B	T1	T2	T3	T1-T3 combined
SC1	0	0	0	0
SC2	0	0	0	0
SC3	0	0	0	0
Non-clonal	9	8	3	20
Non-informative	5	4	2	11
Total number of cells	14	12	5	31
Total number of informative cells	9	8	3	20
Percentage of informative cells	64%	67%	60%	65%
Percentage of informative cells attributable to subclones	0%	0%	0%	0%

T	T1	T2	T3	T1-T3 combined
SC1	0	0	0	0
SC2	4	1	1	6
SC3	1	1	2	4
Non-clonal	192	165	188	545
Non-informative	196	171	273	640
Total number of cells	393	338	464	1195
Total number of informative cells	197	167	191	555
Percentage of informative cells	50%	49%	41%	46%
Percentage of informative cells attributable to subclones	3%	1%	2%	2%

NK	T1	T2	T3	T1-T3 combined
SC1	0	0	1	1
SC2	0	0	0	0
SC3	0	0	0	0
Non-clonal	1	0	0	1
Non-informative	1	0	0	1
Total number of cells	2	0	1	3
Total number of informative cells	1	0	1	2
Percentage of informative cells	50%	na	100%	67%
Percentage of informative cells attributable to subclones	0%	na	100%	50%
