# Supplementary material for: Clonal dynamics of aggressive systemic mastocytosis on avapritinib therapy
Source: Blood Cancer J. 2024 Oct 14;14(1):179. doi: 10.1038/s41408-024-01157-w (PMC11473837; doi:10.1038/s41408-024-01157-w)
Supplement: Supplementary file 9 — Suppl Table 7 Pathology, Cytogenetics, and Next Generation Sequencing [file 41408_2024_1157_MOESM9_ESM.pdf]

Pathology, Cytogenetics, and Next Generation Sequencing

	Diagnosis	Age	Sex	Karyotype	Clinical NGS	
					**Prior to therapy**	**On therapy**
Pt1	ASM-CMML	58	F	46,XX[20]	**Day-166, BM, Montana via Genoptix**<br>TP53 c.541C>T/p.Arg181Cys (27%)<br>SRSF2 c.284C>T/p.Pro95Leu (46%)<br>TET2 c.3764dup/p.Tyr1255 (39%)<br>TET2 c.2211del/p.Leu738fs (39%)<br>RUNX1 c.327_329delCAA/p.Asn109del (11%)<br>RUNX1 c.965C>G, p.S322* (7%)<br>KIT c.2447A>T/p.Asp816Val (6%)	**Day+85, peripheral blood, ARUP**<br>TP53 c.541C>T/p.Arg181Cys (38.8%)<br>SRSF2 c.284C>T/p.Pro95Leu (43.8%)<br>TET2 c.3764dup/p.Tyr1255 (40.4%)<br>TET2 c.2211del/p.Leu738fs (45.8%)<br>RUNX1 c.327_329delCAA/p.Asn109del (3.3%)
Pt2	ASM-MDS	63	F	46,XX[20]	**Day-172, BM, Mayo Clinic**<br>SRSF2 c.282_284delinsTCT/p.Pro95Leu (43%)<br>TE2 c.3400A>T/p.Arg1134* (46%)<br>TE2 c.2501del/p.Cys834fs (44%)<br>KIT c.2447A>T/pAsp816Val (15%)<br>KIT c.1621A>C; p.Met541Leu (48%)-generally considered benign polymorphism	**Day+169, BM, ARUP**<br>SRSF2 c. 282_284delinsTCT/p.Pro95Leu (45.2%)<br>TE2 c.3400A>T/p.Arg1134* (48.2%)<br>TE2 c.2501del/p.Cys834fs (47.4%)
Pt3	ASM-MDS/MPN-U	62	F	46,XX[20]	**Day-119, peripheral blood, ARUP**<br>KIT c.2447A>T/p.Asp816Val (21%)<br>CBL c.1096-1G>T(3%)<br>TET2 c.2737C>T/p.Gln913* (35.3%)<br>TET2 c.2200C>T/p.Gln734* (19%)<br>KDM6A c.2988+1del/c.2988+1del (33.4%)<br>U2AF1 c.445G>A/p.Asp149Asn (14.5%)	**Day+169, peripheral blood, ARUP**<br>KIT c.2447A>T/p.Asp816Val (1.8%)<br>CBL c.1096-1G>T (6.8%)<br>TET2 c.2737C>T/p.Gln913* (42.8%)<br>TET2 c.2200C>T/p.Gln734* (1.7%)<br>KDM6A c.2988+1del/c.2988+1del (32.1%)<br>NRAS c.179G>A/p.Gly60Glu (5%)<br>U2AF1 c.445G>A/p.Asp149Asn (1.7%)
Pt4	ASM-CEL	73	F	46,XX[20]	**Day-827, peripheral blood, ARUP**<br>ASXL1 c.1772dup/p.Tyr591* (34.7%)<br>EZH2 c.2199C>A/p.Tyr733* (55.6%)<br>KIT c.2447A>T/p.Asp816Val (20%)<br>ETNK1 c.731A>G/p.Asn244Ser (39%)<br>EZH2 c.1727G>A/p.Cys576Tyr (23.1%)	**Day+168, peripheral blood, ARUP**<br>ASXL1 c.1772dup/p.Tyr591* (42.5%)<br>EZH2 c.2199C>A/p.Tyr733* (51.9%)<br>KIT c.2447A>T/p.Asp816Val (25.7%)<br>ETNK1 c.731A>G/p.Asn244Ser (40.4%)<br>EZH2 c.1727G>A/p.Cys576Tyr (25.3%)<br>RUNX1 c.352-10T>A(18.2%)
CMML1	CMML	63	F	45,XX, -7[19]/ 46,XX[1]	ND	ND
CMML2	CMML	69	F	46,XX[20]	ND	ND
CMML3	CMML	85	F	46,XX [20]	ND	ND
H1	Healthy	74	M	ND	NA	NA
H2	Healthy	71	M	ND	NA	NA

H3	Healthy	57	F	ND	NA	NA

ND: No data
