# Supplementary material for: Clonal dynamics of aggressive systemic mastocytosis on avapritinib therapy
Source: Blood Cancer J. 2024 Oct 14;14(1):179. doi: 10.1038/s41408-024-01157-w (PMC11473837; doi:10.1038/s41408-024-01157-w)
Supplement: Supplementary file 10 — Suppl Table 8 Primers for sequencing colonies [file 41408_2024_1157_MOESM10_ESM.pdf]

Primers for sequencing colonies

Patient	Variants	Forward Primers	Reverse Primers
P1	TET2 (c.2274delT)	5’-CAACAAAGAGCAGATTCCCAAAC-3’	5’-AGTCTGGCCAAAGAATGATCCTT-3’
P1	TET2 (c.3827dupA)	5’-ACACCTGTGAGGCTGCAGT-3’	5’-TTCTCTCTTCATTCAAGGCACACC-3’
P1	TP53 (c.541C>T)	5’-TGTGGGTTGATTCCACACCCC-3’	5’-CGCAAATTTCCTTCCACTCGGAT-3’
P1	SRSF2 (c.284C>T)	5’-ACCTTTGTGAGGTCGCCCG-3’	5’-AGGCGCGTCTTCGAGAAGTAC-3’
P3	ADH1C (c.232T>G)	5’-AATAACAATTTTAGAAAATTGGGTTTG -3’	5’-TGCCTGAAGTCATACATGCTT-3’
P3	PLK4 (c.694T>A)	5’-ACTACATTTCACCAGAAATTGCCAC-3’	5’-ATTGTGGCATGCCCACTATCA-3’
P3	KIT (c.2447A>T)	5’-GTATTCACAGAGACTTGGC-3’	5’-GTTTCCTTTAACCACATAATTAG-3’
P3	TET2 (c.2263C>T)	5’-CAACAAAGAGCAGATTCCCAAAC-3’	5’-AGTCTGGCCAAAGAATGATCCTT-3’
P3	TAF1 (c.2804C>A)	5’-ACTGCCCTGAGAATCTTTT-3’	5’-CTTGACTTTTTCATTCTAGAAATA-3’
P3	TET2 (c.2800C>T)	5’-AGCAAGATCTTCTTCACAGGTGC-3’	5’-TCAGGCACAGGAAAAACATTTGC-3’
P3	PDE4D (c.90-11229T>C)	5’-TAGTCCCACAGGAATAACTCAGTTT-3’	5’-TTTCCTGTGCAGAAGCTCTTTAG-3’
P4	ASXL1 (c.1772dupA)	5’-CTTTCACGTATCAAACCACCCTG-3’	5’-CTCTATGGCAGTGGTGACCTC-3’
P4	MET (n.105-2delA)	5’-GTTCTGACAGCAGACTGATAACAG-3’	5’-CATGGAATCTCAGAACAAGAAGCCT-3’
P4	ETNK1 (c.731A>G)	5’-AGGCTAATAGCTCGTCAGCT-3’	5’-TTCATCTGCAAATCCTGTGGG-3’
P4	RIT1 (c.204T>G)	5’-ACGGGAGTACAAACTAGTGATGC-3’	5’-ACAACAGACAGACATGGGAGA-3’
P4	DPPA4 (c.673G>A)	5’-TTGTGGTGACAACTTCTGCCC-3’	5’-GCAAATAAATGCTCAGGTCCTG-3’
P4	EZH2 (c.2199C>A)	5’-TTTGTTGTGTTAAGTCTCAGCA -3’	5’-GACAAGTTCAAGTATTCTTTATTCAAAG-3’
P4	KIT (c.2447A>T)	5’-GTATTCACAGAGACTTGGC-3’	5’-GTTTCCTTTAACCACATAATTAG-3’
P4	EZH2 (c.1727G>A)	5’-CAAAACCGCTTTCCGGGATG-3’	5’-TGCAGTTCTTGCAGGACACATT-3’
P4	CD163L1 (c.2732G>A)	5’-AGTTGGAGTTGTCTGTTCCCG-3’	5’-GCTGAGCTGTCTGCATAGAACAC-3’
