# Supplementary material for: Clonal dynamics of aggressive systemic mastocytosis on avapritinib therapy
Source: Blood Cancer J. 2024 Oct 14;14(1):179. doi: 10.1038/s41408-024-01157-w (PMC11473837; doi:10.1038/s41408-024-01157-w)
Supplement: Supplementary file 11 — Suppl Table 9 Markers for cell type identification [file 41408_2024_1157_MOESM11_ESM.pdf]

Markers for cell type identification

Gene	Cell type
MS4A1	B cell
NKG7	NK
GNLY	NK
CD8A	CD8+ T
CD4	CD4+ T
CD3E	T
CD14	Monocyte
FCGR3A	CD16+ monocyte
FCER1A	DC
FCGR3B	Neutrophil
PPBP	Plt
HBB	RBC
CCR3	Basophil
CLC	Basophil
HDC	Basophil
KIT	Mast cell
TPSAB1	Mast cell
CPA3	Mast cell
ITGAM	Mature neutrophil
CEACAM3	Mature neutrophil
CEACAM1	Immature neutrophil
CEACAM8	Immature neutrophil
LAIR1	Eosinophil
ITGA4	Eosinophil
IL3RA	Eosinophil
COL1A1	mesenchymal cell
COL1A2	mesenchymal cell
COL3A1	mesenchymal cell
CD79A	plasma cell
MBZ1	plasma cell
